# Analysis of Genetic Interactions Involving Maternal and Offspring Genotypes at Different Loci: Power Simulation and Application to Testicular Cancer

**DOI:** 10.1002/gepi.21655

**Published:** 2012-06

**Authors:** Jérémie Nsengimana, Jennifer H Barrett

**Affiliations:** Section of Epidemiology and Biostatistics Leeds Institute of Molecular Medicine University of LeedsLeeds United Kingdom

**Keywords:** family trios, EM algorithm, two-locus log-linear model, type 1 error

## Abstract

The analyses of genetic interaction between maternal and offspring genotypes are usually conducted considering a single locus. Here, we propose testing maternal × offspring (M×O) and maternal × maternal (M×M) genotype interactions involving two unlinked loci. We reformulate the log-linear approach of analyzing cases and their parents (family trios) to accommodate two loci, fit fuller models to avoid confounding in a first analysis step and propose that the model be reduced to the most prominent effects in a second step. We conduct extensive simulations to assess the validity and power of this approach under various model assumptions. We show that the approach is valid and has good power to detect M×O and M×M interactions. For example, the power to detect a dominant interaction relative risk of 1.5 (both M×O and M×M) is 70% with 300 trios and approaches 100% with 1,000 trios. Unlike the main effects, M×O and M×M interactions are conditionally independent of mating types, and consequently, their power is not affected by missing paternal genotypes. When applied to single-locus M×O interaction, our method is as powerful as other existing methods. Applying the method to testicular cancer, we found a nominally significant M×M interaction between single nucleotide polymorphisms from C-Kit Ligand (*KITLG*) and Sex Hormone Binding Globulin (*SHBG*) using 210 families (relative risk 2.2, *P* = 0.03). This finding supports a role of maternal hormones in offspring testicular cancer and warrants confirmation in a larger dataset.

## INTRODUCTION

It has been suggested that maternal effects play an important role in species adaptation [Wolf, 2000], and there is a compelling body of evidence that complex adulthood diseases, such as diabetes, hypertension, schizophrenia, asthma, testicular, renal, and gastric cancers, are partially determined by antenatal factors [Drever et al., 2010; Eriksson et al., 2011; Maringhini et al., 2010; Winder et al., 2011]. Although the process appears to be dominated by in utero epigenetic changes, placental size, and foetal growth rate, all of which are directly related to maternal diet [Barker et al., 2010], maternal genes involved in placental formation, adhesion, vascularization, hormone secretion, or transport activity are also likely to be involved [Jansson et al., 2002].

Dissecting maternal effects is attracting renewed attention, and novel statistical methods and designs, such as those exploiting Assisted Reproduction Technology (ART), are emerging [Rice et al., 2009; Thapar et al., 2007; Zhou et al., 2011]. ART allows complete separation of the effects of the child's genotype from those of the maternal genotype when gestational and biological mothers are different. More classical family designs have also been used to jointly analyze these effects [Weinberg et al., 1998], with the possibility to include maternal-foetal interaction at one locus [Ainsworth et al., 2011; Sinsheimer et al., 2003]. Effects that are seldom analyzed are the interaction between a maternal genotype at one locus and a child genotype at another locus, and the interaction of two maternal loci to raise the disease risk in their offspring. Maternal × offspring (M×O) interaction at different loci is ubiquitous in mammals [Wolf, 2000]. In a mouse model, it was shown that maternal thyroid hormones bind the embryonic thyroid receptors [Nucera et al., 2010] and a list of 81 foetal genes have been identified that are maternally regulated [SenthamaraiKannan et al., 2011]. M×O interactions may partially determine birth-weight, a trait associated with many adulthood diseases [Wolf, 2000]. Simulation studies have shown that when M×O interaction exists, the maternally expressed allele may persist longer at a higher frequency in the population than the zygotically expressed allele, and under certain genetic models, it escapes from the purifying selection, increasing the disease prevalence in the population [Priest and Wade, 2010]. An interaction between two maternal genes (M×M interaction) can also increase the child disease risk. For example, an association was found between craniofacial malformations and an interaction between maternal single nucleotide polymorphism (SNP) genotypes in folate and choline metabolism genes [Mostowska et al., 2010a, b].

The risk of developing testicular germ cell tumor (TGCT) has long been associated with antenatal exposure to maternal hormones. When exposed to high levels of estrogens in vitro, primordial germ cells (PGCs, the embryonic precursors of gametes) start transcription of C-kit ligand (*KITLG*), and the accumulation of this growth factor eventually transforms PGCs into tumorigenic cells [Moe-Behrens et al., 2003]. We and others have found that SNPs from *KITLG* are the most associated with TGCT in genome-wide association studies (GWAS) [Kanetsky et al., 2009; Rapley et al., 2009] and it can be hypothesized that the disease-initiating events occur in the early embryonic development from an interplay between maternal estrogens and child or maternal *KITLG*. The purpose of this study is to investigate the application of log-linear methods to test the interactions between a maternal genotype at one locus and a child genotype at another unlinked locus and between two unlinked maternal loci. We use a method originally proposed to analyze gene-environment interactions [Umbach and Weinberg, 2000], considering as exposure a second locus maternal genotype. Our study extends and investigates this approach in several ways: first, because genetic effects from maternal and child genotypes can be mutually confounded, we fit a model including the main effects from child and maternal genotypes along with two-locus M×O and M×M interactions. We propose that a further analysis stage may be conducted, where the least significant effects are dropped from the model and we discuss the implications of this model selection process. Second, we simulated data to assess the relative merits (power and type 1 error) of different approaches to testing for M×O and M×M interactions: testing assuming mating symmetry, either specifying or estimating the second locus genotype frequency distribution, and testing assuming Hardy-Weinberg equilibrium (HWE). Third, we investigate the sensitivity of the M×O and M×M interactions test to the violation of the assumption of parental mating symmetry. Fourth, we compare the power to detect the interactions to the power to detect main effects. Finally, we use the Expectation-Maximization (EM) algorithm to analyze incomplete trios and show that M×O and M×M interaction tests are independent of missing paternal genotypes. Therefore, although the methods considered in this study have been used previously, we revisit them from a different angle, reformulating the log-linear framework to accommodate two-locus models and exploring various novel aspects before conducting the power simulations. We then apply the method to a TGCT dataset, examining the interaction between a maternal hormone-related gene and *KITLG*.

## METHODS

### TWO-LOCUS LOG-LINEAR MODEL

We assume that family trios have been genotyped at a first locus and that the mothers are additionally genotyped at a second locus, both unlinked and in linkage equilibrium with the first locus. Although some of the tests we propose rely only on the assumption of linkage equilibrium, M×O and M×M cannot be tested simultaneously without imposing the absence of both linkage and linkage disequilibrium (LD). Therefore, throughout this article when we say “unlinked” we also imply absence of LD. Originally, the log-linear method was designed to analyze genetic effects from one locus in cases, their mothers, or both, and possibly subject to imprinting [Weinberg et al., 1998]. Under the assumption of mating symmetry, joint genotypes of parents define six mating types, and joint genotypes of a family trio define 15 categories (see Table I). If mothers of cases are additionally genotyped at a second locus (*M*_2_), the 15 categories can be stratified on the second locus genotypes and the proportions of family trios falling in each of the new defined categories is given in Table I. Using the original notation of Weinberg et al. [1998], locus 1 has main maternal effects *S_i_* and main offspring effects *R_j_*, where *i*, *j* represent the number of susceptibility alleles carried by mother and child, respectively. *S_i_* and *R_j_* are the relative excess of disease risk in the offspring conferred by maternal and offspring disease alleles. Scaling factors μ*_m_* are population frequencies of the mating types.

**TABLE I:** Theoretical proportions of family trio types in a two-locus model

					Expected cell proportions							
Configuration	Mating type	*M*^a^	*F*^a^	*C*^a^	*M*_2_ = 0^b^	*M*_2_ = 1^b^	*M*_2_ = 2^b^
1	1	2	2	2	*p*_0_*S*_2_*R*_2_μ_1_	*p*_1_ϑ_21_Φ_21_*S*_2_*R*_2_μ_1_τ_1_	*p*_2_ϑ_22_Φ_22_*S*_2_*R*_2_μ_1_τ_2_
2	2	2	1	2	*p*_0_*S*_2_*R*_2_μ_2_	*p*_1_ϑ_21_Φ_21_*S*_2_*R*_2_μ_2_τ_1_	*p*_2_ϑ_22_Φ_22_ *S*_2_*R*_2_μ_2_τ_2_
3	2	2	1	1	*p*_0_*S*_2_*R*_1_μ_2_	*p*_1_ϑ_21_Φ_11_*S*_2_*R*_1_μ_2_τ_1_	*p*_2_ϑ_22_Φ_12_*S*_2_*R*_1_μ_2_τ_2_
4	2	1	2	2	*p*_0_*S*_1_*R*_2_μ_2_	*p*_1_ϑ_11_Φ_21_*S*_1_*R*_2_μ_2_τ_1_	*p*_2_ϑ_12_Φ_22_*S*_1_*R*_2_μ_2_τ_2_
5	2	1	2	1	*p*_0_*S*_1_*R*_1_μ_2_	*p*_1_ϑ_11_Φ_11_*S*_1_*R*_1_μ_2_τ_1_	*p*_2_ϑ_12_Φ_12_*S*_1_*R*_1_μ_2_τ_2_
6	3	2	0	1	*p*_0_*S*_2_*R*_1_μ_3_	*p*_1_ϑ_21_Φ_11_*S*_2_*R*_1_μ_3_τ_1_	*p*_2_ϑ_22_Φ_12_*S*_2_*R*_1_μ_3_τ_2_
7	3	0	2	1	*p*_0_*R*_1_μ_3_	*p*_1_Φ_11_*R*_1_μ_3_τ_1_	*p*_2_Φ_12_*R*_1_μ_3_τ_2_
8	4	1	1	2	*p*_0_*S*_1_*R*_2_μ_4_	*p*_1_ϑ_11_Φ_21_*S*_1_*R*_2_μ_4_τ_1_	*p*_2_ϑ_12_ Φ_22_*S*_1_*R*_2_μ_4_τ_2_
9	4	1	1	1	*2p*_0_*S*_1_*R*_1_μ_4_	2*p*_1_ϑ_11_Φ_11_*S*_1_*R*_1_μ_4_τ_1_	2*p*_2_ϑ_12_Φ_12_*S*_1_*R*_1_μ_4_τ_2_
10	4	1	1	0	*p*_0_*S*_1_μ_4_	*p*_1_ϑ_11_*S*_1_μ_4_τ_1_	*p*_2_ϑ_12_*S*_1_μ_4_τ_2_
11	5	1	0	1	*p*_0_*S*_1_*R*_1_μ_5_	*p*_1_ϑ_11_Φ_11_*S*_1_*R*_1_μ_5_τ_1_	*p*_2_ϑ_12_Φ_12_*S*_1_R_1_μ_5_τ_2_
12	5	1	0	0	*p*_0_*S*_1_μ_5_	*p*_1_ϑ_11_*S*_1_μ_5_τ_1_	*p*_2_ϑ_12_*S*_1_μ_5_τ_2_
13	5	0	1	1	*p*_0_*R*_1_μ_5_	*p*_1_Φ_11_*R*_1_μ_5_τ_1_	*p*_2_Φ_12_*R*_1_μ_5_τ_2_
14	5	0	1	0	*p*_0_μ_5_	*p*_1_μ_5_τ_1_	*p*_2_μ_5_τ_2_
15	6	0	0	0	*p*_0_μ_6_	*p*_1_μ_6_τ_1_	*p*_2_μ_6_τ_2_

^a^M, F, C: maternal, paternal, and child genotypes at the first locus.

^b^*M*_2_ = maternal genotypes at the second locus with frequency *p_k_*, *k* = 0,1,2. Genotypes are coded as the number of minor alleles (assumed to increase the risk).

We introduce two new sets of parameters: ϑ*_ik_* coefficient for M×M interaction between the two loci and Φ*_jk_* for M×O interaction (index *k* is the number of maternal alleles at locus 2, assumed to be unlinked to locus 1). Stratifying the expected cell counts by the second locus implies that each of the new cells must be weighted by the population genotype frequencies *p_k_* at this locus. Using Table I, a two-locus log-linear model of family trio counts can be written as:

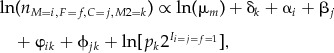
where *n_M_*_= *i*,*F* = *f*,*C* = *j*,*M*2 = *k*_ is the expected number of family trios with specific maternal (*M*), paternal (*F*), and child (*C*) genotype at locus 1 and maternal (*M*_2_) genotype at locus 2. Mating type index *m* on the right-hand side of the equation ranges from 1 to 6 as (*M*,*F*) on the left-hand side takes the values (2,2), (2,1), (2,0), (1,1), (1,0), and (0,0). Regression coefficients α*_i_*, β*_j_*, ϕ*_ik_*, and φ*_jk_* are, respectively, the natural logarithm of disease relative risks *S_i_*, *R_j_*, ϑ*_ik_*, and Φ*_jk_* of Table I. All these coefficients are set to zero for the baseline genotype, i.e., α_0_ = β_0_ = 0, ϕ*_ik_* = 0 if *i* = 0 or *k* = 0, and φ*_jk_* = 0 if *j* = 0 or *k* = 0. The last term of the equation represents a “weight” or “offset.” There are two different ways giving rise to a heterozygous offspring when both parents are heterozygous, and they are equally likely in the absence of imprinting. In this case, the cell count is weighted by a factor of 2, which is reflected in the “offset” (i.e., the indicator variable *I_i_*_= *j* = *f* = *1*_ takes the value of 1). In addition, with a second locus, each cell count is proportional to the genotype frequencies *p_k_* as shown in Table I, and this is also reflected in the “offset” term.

Parameter δ*_k_* is the natural logarithm of τ*_k_* shown in Table I, which fits the main effect of maternal genotype *k* at locus 2, assuming the absence of effect from the baseline genotype (δ_0_ = 0). Although we are not interested in this effect, its inclusion in the model is necessary to ensure that the interaction test does not depend on genotype frequencies. On closer inspection, it can be seen that each of the new cells created by stratifying on locus 2 is weighted by genotype frequencies independently of coefficients ϑ*_ik_* and Φ*_jk_* (Table I). It follows that if one were interested in testing the locus 2 main effect, knowing its frequency distribution with precision would be required. It would also be required if one fitted the interaction terms without this main effect in the model. However, as we will show in simulations, estimates of the interaction terms are independent of the genotype frequencies provided that the main effect is included. Furthermore, Equation (1) is valid for testing the main effect δ*_k_* itself when the genotype frequencies are correctly specified in the offset term (see Results).

Equation (1) requires specifying the second locus genotype frequencies in a similar way to the environmental risk factor ratios used by Umbach and Weinberg [2000] (see their equation on p. 257). However, as we consider a three-level exposure (second locus genotype), we fit a two-level main effect (δ*_k_*, *k* = 1,2) instead of one level. Their term, which is the natural logarithm of the ratio of exposed to unexposed individuals in the population, would be written as ln(*p_k_/p*_0_) in our notation. The Umbach and Weinberg [2000] term μ*_i_* is then equivalent to ln(μ*_m_*) + ln(*p*_0_) in our notation.

The locus 2 genotype frequencies could be estimated, instead of specifying them in an offset term as in Equation (1). However, now the second locus main effect cannot be estimated. Equation (2) describes this model: 

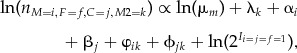
where λ*_k_* equals ln(*p_k_/p*_0_), *k* = 1,2. Using the ratio of frequencies in the model ensures that their sum is constrained to equal to 1.

An alternative approach to reduce the number of parameters is to use Equation (1) and assume HWE for both loci. In fact, μ*_m_* can be expressed as a function of one allele frequency if HWE is assumed at locus 1 [Weinberg et al., 1998]. However, unlike the main effects *S_i_* and *R_j_* that depend directly on the mating types, estimates of the interaction terms ϑ*_ik_* and Φ*_jk_* are conditionally independent of these nuisance parameters when *S_i_* and *R_j_* are fitted in the model. To see this, note that in Table I the interaction coefficients ϑ*_ik_* and Φ*_jk_* are defined in columns where *M*_2_ = 1 and *M*_2_ = 2 and they are tested against the baseline column *M*_2_ = 0. They are independent from mating type strata because the same coefficient μ*_m_* multiplies cell counts in these three columns on each row of Table I. Therefore, modeling all mating types or replacing them by one parameter does not affect the power to detect the interactions (see Results).

Using Equation (1) or Equation (2), M×M and M×O interactions can be tested in a likelihood ratio test of the full model including all the effects vs. a model excluding the effect investigated. Log-linear methods also allow the analysis of M×O interactions involving one locus (coefficient ρ*_ij_* in Equation (3), defined for *i* > 0 and *j* > 0, set to zero if *I* = 0 or *j* = 0). A likelihood ratio test of the interaction term ρ*_ij_* is equivalent to the multinomial test used in the EMIM software [Ainsworth et al., 2011] and to the method of Sinsheimer et al. [2003] implemented in the software package Mendel [Lange et al., 2001]. 

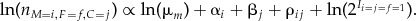


### INCORPORATING INCOMPLETELY GENOTYPED TRIOS

The EM algorithm is a powerful approach to jointly analyzing full trios and incompletely genotyped trios in the log-linear framework [Weinberg, 1999]. For the analysis of M×M and M×O interactions, tests from Equation (1) and (2) are completely immune to missing paternal genotypes. This is because, as said earlier, the interaction terms ϑ*_ik_* and Φ*_jk_* are conditionally independent of the mating type strata when *S_i_* and *R_j_* are included in the model. In other words, missing fathers cause an ambiguity of mating types that could have an impact on the power to detect main effects *S_i_* and *R_j_*, but the interactions ϑ*_ik_* and Φ*_jk_* are completely unaffected. However, when the missing parent is the mother, it is reasonable to assume that her genotypes will be unavailable for both loci, in which case the data contain little information about locus 2. We investigate the method's efficiency for M×M and M×O interactions when the EM algorithm is used to analyze trios with missing parental genotypes. More information on the application of the EM algorithm to incomplete family trios can be found in Weinberg [1999].

## SIMULATIONS

We designed simulations to evaluate the type 1 error and power of Equation (1) and (2) to test two-locus M×M and M×O interactions as well as (3) for single-locus M×O interaction. In all simulations, we considered one locus genotyped in family trios (locus 1) and another, unlinked and in linkage equilibrium in the general population, genotyped only in the mothers (locus 2). In all simulated models, 1,000 replicates were generated where the baseline disease incidence rate was 0.1, for both loci HWE was assumed and the risk allele frequency (RAF) was 0.3. Under dominant inheritance (Table II), we simulated one example of a small dataset (300 family trios) and one moderate sample size (1,000 family trios). Only the sample size of 1,000 trios was considered for codominant genetic inheritance (Table III). Some of the simulated models were intended to assess type 1 error or power for both M×M and M×O interactions, while other models were designed to assess the type 1 error for one type of interaction and the power for the other. These models therefore allow possible confounding between the two effects to be assessed. They also allow an evaluation of the power gain in models restricted to only the genuine effects compared to a more saturated model. Models 11a–14a (Table III) were simulated under parental mating asymmetry to assess the sensitivity of the interaction tests to violation of the mating symmetry assumption. In these models, locus 1 RAF was 0.3 in females, but two equally sized strata of males were considered: one with RAF 0.1 and another with RAF 0.5, with HWE within each stratum. This corresponds to an overall RAF of 0.3 for males in the combined population, but gives rise to asymmetry in the mating patterns, since, for example, a female is now more likely to be heterozygote than is a male. For locus 2, the RAF remained 0.30 in females and in each of the two male strata, and the two loci were unlinked as before.

**TABLE II:** Simulated dominant models to evaluate the interaction tests

Model	*R*	*S*	ρ	exp(*δ*)	ϑ	Φ	Purpose^a^
1	1	1	1	1	1	1	Type 1 error of Equation (1) and (2) assuming mating symmetry, (1) assuming HWE, full data and missing parents, robustness to frequency misspecification.
2	1	1	1	1	1.5	1	Power + type 1 error.
3	1	1	1	1	2	1	Power + type 1 error.
4	1	1	1	1	1	1.5	Power + type 1 error.
5	1	1	1	1	1	2	Power + type 1 error.
6	1.5	1.5	1	1	1	1	Type 1 error + power for main effects of child and maternal genotypes, estimation of genotype frequencies with Equation (2)
7	1	1	1	1	1.5	1.5	Power comparison Equation (1) vs. Equation (2), full data and missing parents, estimation of genotype frequencies with Equation (2)
8	1	1	1	1	2	2	Power comparison Equation (1) vs. Equation (2), full data and missing parents, estimation of genotype frequencies with Equation (2)
9	1	1	1.5	1	1	1	Power comparison Equation (3) vs. EMIM.
10	1	1	2	1	1	1	Power comparison Equation (3) vs. EMIM.

^a^*R*, *S*, Φ, ϑ, and δ represent, respectively, the main effect of child locus 1, main effect of maternal locus 1, two-locus M×O interactions, two-locus M×M interactions, and maternal second locus main effect. The purpose of simulations was to evaluate the power and/or type 1 error to detect M×M and M×O interactions in 300 and 1,000 full trios using Equation (1), assuming mating symmetry and correctly specifying locus 2 genotype frequencies, unless otherwise indicated. Dominant models are simulated for all relevant effects, i.e., *R* = *R*_1_ = *R*_2_, *S* = *S*_1_ = *S*_2_, ρ = ρ_11_ = ρ_12_ = ρ_21_ = ρ_22_, ϑ = ϑ_11_ = ϑ_12_ = ϑ_21_ = ϑ_22_, and Φ = Φ_11_ = Φ_12_ = Φ_21_ = Φ_22_ and locus 2 has no main maternal effect (δ_1_ = δ_2_ = 0). The data simulated in these models were analyzed assuming dominant inheritance of all the effects tested and two nuisance parameters δ_1_ and δ_2_ were fitted for the main maternal effect of the second locus.

**TABLE III:** Simulated codominant models^a^

Model	*R*_1_	*R*_2_	*S*_1_	*S*_2_	Φ_11_	Φ_12_	Φ_21_	Φ_22_	ϑ_11_	ϑ_12_	ϑ_21_	ϑ_22_	δ_1_	δ_2_
11 + 11a	1	1	1	1	1.3	1.69	1.69	2.197	1	1	1	1	1	1
12 + 12a	1	1	1	1	1	1	1	1	1.4	1.96	1.96	2.744	1	1
13 + 13a	1	1	1	1	1.3	1.69	1.69	2.197	1.4	1.96	1.96	2.744	1	1
14 + 14a	1.3	1.69	1.4	1.96	1	1	1	1	1	1	1	1	1	1

^a^*R_j_*, *S_i_*, *Φ_jk_*, and *ϑ_ik_* and *δ_k_* represent, respectively, the main effect of child locus 1, main effect of maternal locus 1, two-locus M×O interactions, two-locus M×M interactions, and maternal second locus main effect. Models 11–14 were simulated under parental mating symmetry while 11a–14a were simulated under parental mating asymmetry with similar relative risks. Data simulated under these models were analyzed using Equation (1) assuming an unconstrained genetic model for all parameters of interest (*S_i_*, *R_j_*, Φ*_jk_*, and ϑ*_ik_*) along with nuisance parameters δ_1_ and δ_2_. Robustness to genetic model misspecification was assessed by repeating the analysis assuming dominant effects in the analysis.

To demonstrate the independence of interaction tests from genotype frequency specification, datasets simulated under model 1 (see Table II) were analyzed assuming a broad range of the locus 2 genotype frequency distributions. To illustrate the immunity of the interaction tests to missing paternal data, we generated missing paternal genotypes in 10–80% of families in model 1 simulations. To assess the ability of the EM algorithm to handle missing data for two loci, we simulated missing maternal genotypes in 5–30% of families. Using data simulated under parental mating symmetry, we further compare: (1) the power of detecting two-locus M×M and M×O interactions using Equation (1) and (2), both assuming mating symmetry and Equation (1) assuming HWE (Equation (1)_hwe); (2) the power to detect these interactions vs. the power to detect the main effects *S_i_* and *R_j_* of similar magnitude; and (3) the power to detect single-locus M×O interaction using Equation (3) vs. EMIM. The analysis including the main child and maternal effects from locus 1, a main effect from locus 2 and the two-locus M×O and M×M interactions under dominance or codominance will be referred to as a full model, while the analysis restricted to a subset of these effects will be referred to as a reduced or restricted model. Although our primary focus is on the validity and power of the interactions tests, we also report as supplementary data the risk parameter estimates for the simulated models. All data were simulated with a purpose-built C program and analyzed in STATA using Poisson regression. These programs are available from the authors on request.

## APPLICATION TO TGCT

We applied two-locus log-linear models to 210 families (147 full trios and 63 case-mother pairs), testing the interactions between *KITLG* SNPs (rs1508595 and rs995030, each separately) and rs6259, a missense SNP from Sex Hormone Binding Globulin (*SHBG*; it binds estrogens and other sex hormones, regulating their availability). The two *KITLG* SNPs were the most significant in a TGCT GWAS and showed independent signals in multivariable analysis (Rapley et al., 2009). The LD between *KITLG* SNPs was estimated using 640 subjects (370 case parents and 270 unrelated controls) with STATA add-on command *pwld*.

## RESULTS

### VALIDITY AND POWER OF TWO-LOCUS INTERACTION TESTS IN FULL TRIOS

Table IV summarizes the type 1 error and power to detect M×M and M×O interactions at the nominal significance level of 0.05 in different simulated datasets with 300 or 1,000 trios. There are five sections in the table: the top section shows for simulated models 1 and 6 the type 1 error of the interactions using Equations (1) or (2), both assuming mating symmetry. For model 1, this section further depicts the robustness of (1) to misspecifying the second locus genotype frequencies and assesses the type 1 error of (1) under the assumption of HWE at locus 1 (Equation (1)_hwe). All the results in this section show that the interaction tests have a non-inflated type 1 error using all the methods. The highest error observed is 5.8%, which is not significantly higher than 5% (one-sided *P*-value for the difference is 0.14). Results also confirm that the interaction tests based on Equation (1) are robust to genotype frequency misspecification: data analysis assuming a frequency distribution of 0.01, 0.18, 0.81 as well as 0.25, 0.50, 0.25 gave exactly the same interaction error rate as when the correct genotype frequencies of 0.09, 0.42, and 0.49 were used. As explained earlier, this is because the uncertainty is compensated for by the inclusion of the second locus main effect. However, it is important to note that this main effect can only be accurately estimated if the genotype frequency distribution is correctly specified (see next paragraph). Model 6 results show that main and interaction effects are not confounded if they are all fitted.

**TABLE IV:** Power and type 1 error (%) of the interactions using full trios (α = 0.05)

Simulated Model^a^	No. families	Method^b^	Assumed *p_k_* if Equation (1)^c^	ϑ^d^	Φ^d^
1	300	Equation (1)	True	4.8	4.6
1	300	Equation (1)	False 1	4.8	4.6
1	300	Equation (1)	False 2	4.8	4.6
1	300	Equation 1_hwe	True	4.5	4.7
1	300	Equation (2)	-	4.8	4.6
1	1,000	Equation (1)	True	4.4	3.8
1	1,000	Equation (1)	False 1	4.4	3.8
1	1,000	Equation (1)	False 2	4.4	3.8
1	1,000	Equation 1_hwe	True	4.2	3.8
1	1,000	Equation (2)	-	4.4	3.8
6	300	Equation (1)	True	5.2	5.7
6	300	Equation (2)	-	5.2	5.7
6	1,000	Equation (1)	True	5.8	5.4
6	1,000	Equation (1)	-	5.8	5.4
2	300	Equation (1)	True	32.7	4.3
3	300	Equation (1)	True	73.6	4.5
4	300	Equation (1)	True	5.1	34.6
5	300	Equation (1)	True	5.2	73.9
2	1,000	Equation (1)	True	81.9	5.5
3	1,000	Equation (1)	True	99.5	5.3
4	1,000	Equation (1)	True	5.7	82.2
5	1,000	Equation (1)	True	5.4	99.6
7	300	Equation (1)	True	31.9	34.4
7	300	Equation 1_hwe	True	32.1	34.4
7	300	Equation (2)	-	31.9	34.4
8	300	Equation (1)	True	65.0	66.6
8	300	Equation 1_hwe	True	65.9	67.5
8	300	Equation (2)	-	65.0	66.6
7	1,000	Equation (1)	True	78.2	79.2
7	1,000	Equation 1_hwe	True	78.7	79.3
7	1,000	Equation (2)	-	78.2	79.2
8	1,000	Equation (1)	True	99.0	99.0
8	1,000	Equation 1_hwe	True	99.3	99.0
8	1,000	Equation (2)	-	99.0	99.0
11	1,000	Equation (1)	True	4.8	60.6
12	1,000	Equation (1)	True	84.9	5.0
13	1,000	Equation (1)	True	85.5	61.0
14	1,000	Equation (1)	True	4.9	5.1
11a	1,000	Equation (1)	True	4.5	63.5
12a	1,000	Equation (1)	True	84.5	4.3
13a	1,000	Equation (1)	True	85.4	62.3
14a	1,000	Equation (1)	True	4.0	4.6

^a^Each model was simulated in one run with 300 trios and in a separate run with 1,000 trios, each repeated 1,000 times, and the data were analyzed using the approaches in column 3 in a full model assuming dominant (models 1–8) or codominant inheritance (models 11–14 and 11a–14a).

^b^Equation (1) and (2) assume mating symmetry, Equation (1)_hwe assumes HWE at locus 1. Genotype frequencies estimated with Equation (2) are supplied in Supplementary Material Table S1.

^c^The true genotype frequency distribution simulated was 0.09/0.42/0.49 for the minor homozygous/heterozygous/major homozygous. False 1 and False 2 distributions assumed in some analyses were, respectively, 0.01/0.18/0.81 and 0.25/0.50/0.25.

^d^Power or type 1 error in same column; see simulated models in Tables II and Tables III. Φ and ϑ are M×O and M×M interactions, respectively.

The second section of Table IV shows models 2–5 where one type of interaction was simulated under the null while the other was under the alternative hypothesis. The results in this section show that the interaction tests are always valid and have reasonable power. For example, in simulation model 3 with 300 trios, the M×O test has a type 1 error rate of 4.5% and the M×M test has a power of 73.6% at a nominal 5% significance level. The third section of Table IV shows the power for both M×M and M×O interactions (models 7 and 8) using Equations (1), (1)_hwe, and (2). In all simulated models and sample sizes, Equations (1) and (2) have the same power to detect the interactions, since the only difference between them is in the treatment of the confounded main effects and genotype frequencies, and Equation 1_hwe shows similar power. For example, in model 8 with 300 families, the powers are 65.0% and 66.6% for the two interactions with (1) and (2), while they are 65.9% and 67.5% with (1)_hwe. Results in the fourth section of Table IV (models 11–14) were obtained assuming an unconstrained genotypic model estimating 12 different effects and eight nuisance parameters. They show that M×O and M×M tests remain valid and retain good power with 1,000 full trios. Results in the last section of Table IV show that two-locus interaction tests remain valid and do not lose power when true parental mating is asymmetric but symmetry is assumed in the analysis.

Supplementary Material Table S1 shows that the frequencies of the second locus genotypes are accurately estimated with Equation (2), although this is based on the assumption that the second locus has no main effect. Risk parameter estimates using Equation (1) are unbiased and generally close to their true values (Tables S2–S5 in Supplementary Material). These risk estimates remain accurate when parental mating asymmetry is simulated but symmetry is assumed in the analysis.

### POWER FOR MAIN EFFECTS AND SINGLE-LOCUS M×O INTERACTION

**Figure 1:**
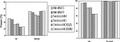
Power (α = 0.05) to detect different types of genetic effects with similar strength (relative risk 1.5) using full and restricted models. The restricted model fits the truly simulated effects plus the second locus main effect when there is an interaction involving this locus. Data from simulated model 6 for *R* and *S*, model 2 for M×M interaction, model 4 for two-locus M×O interaction and model 9 for single-locus M×O interaction. Left panel: 300 full trios; right panel: 1,000 full trios. Analyses conducted using Equation (1) unless otherwise indicated. The analysis assumed a dominant genetic model.

Although assuming HWE does not improve the power to detect M×M and M×O interactions (see Table IV), it does improve the power for the main effects as shown in Supplementary Material Table S6 (model 6). Overall, the power to detect the main effects is substantially higher than the power to detect interactions. As has been reported by others [Sinsheimer et al., 2003], we found that assuming mating type symmetry in testing the main child effect remains valid when the mating is asymmetric while testing the main maternal effect is invalid (Table S6 in Supplementary Material, model 13a). We stated in the Methods section that Equation (1) could be used to test the second locus main effect when its genotype frequency distribution is known, and Supplementary Material Table S7 gives a confirmation of this statement. However, a small deviation from the correct frequency can cause considerable effect inflation, invalidating the test of the main effect. Using the data simulated in model 1, we fitted Equation (3) to assess its type 1 error for single-locus M×O interaction. The type 1 error found at level 0.05 was 5.2% with 1,000 trios and 5.5% with 300 trios. With sample sizes 300 and 1,000 trios, we found that Equation (3) and EMIM have similar power to detect single-locus M×O interactions (Fig. 1).

### MODEL REDUCTION

All the results presented so far were obtained when fitting a full model. While this full model is necessary to detect all the existing effects, the large number of terms impacts on the power to detect each of them. Restricting the analysis to the truly simulated effects (in addition to locus 2 main effect terms when there is an interaction involving this locus), the power is substantially increased, as can be seen in Figure.

### TYPE 1 ERROR AND POWER FOR THE INTERACTIONS WITH MISSING PARENTS

The validity, power, and estimation accuracy of the M×M and M×O interaction tests using Equation [Bibr b1] are not affected by missing fathers up to 80% (Figs. and  ; Table S2 in Supplementary Material). By contrast, when genotypes are missing in mothers of cases at the two loci considered, both interaction tests have an inflated type 1 error. In an initial sample size of 300 trios, there is a significant inflation with 10% missing mothers or more, while this occurs with 15% or more missing mothers in an initial sample size of 1,000 trios (Fig.). With a total sample size of 1,000 families, power to detect M×M and M×O interactions is unaffected by up to 10% missing mothers (Table S8 in Supplementary Material), while their risk parameter estimates remain accurate with up to 20% missing mothers (Table S2 in Supplementary Material). When parental mating asymmetry is simulated but the symmetry is assumed in the analysis, as much as 80% missing fathers do not affect the validity, the power, or the estimation accuracy of the two-locus interactions test (Tables S5 and S9 in Supplementary Material).

**Figure 2:**
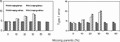
Type 1 error rate for M×M and M×O interactions at nominal level 0.05 with missing parents. Left panel: total sample of 300 families; right panel: total sample of 1,000 families. Data from simulated model 1 and analyzed using the full model (1). The analysis assumed a dominant genetic model.

**Figure 3:**
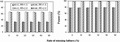
Power for M×M and M×O interactions at nominal level 0.05 with 10–80% missing fathers. Left panel: total sample of 300 families; right panel: total sample of 1,000 families. Data from simulated models 7 and 8 and analyzed using the full model (1). The analysis assumed a dominant genetic model.

### ROBUSTNESS TO MODEL MISSPECIFICATION

When the correct model for the interactions is codominant, assuming a dominant model in the analysis does not invalidate the test, nor does it cause a power loss (Table S10 in Supplementary Material). However, as expected, the parameters estimation becomes inaccurate when the effects exist (Table S11 in Supplementary Material).

### TGCT DATASET ANALYSIS

Despite showing independent effects on TGCT in GWAS, we found that *KITLG* rs1508595 and rs995030 are in strong LD (*D*′ = 0.91, *r*^2^ = 0.65, MAF = 0.17 and 0.14, respectively). In the original results from the GWAS [Kanetsky et al., 2009; Rapley et al., 2009], where major rather than minor alleles were tested, allelic relative risks were estimated to be 3.1 and 2.6, respectively, and the pattern of risk was consistent with a multiplicative genetic model. In view of the small sample size and rarity of the minor homozygote genotype, we here carry out a primary analysis of the 147 full trios and 63 case-mother pairs assuming dominant main effects and interactions using Equation (1) in full and reduced models. Case-father pairs were not used since simulations showed that they could inflate type 1 error. The reduced model included the two most prominent effects in the full model. Table V reports the results: in the full model, the most significant effect was the inherited *KITLG* genotypes. Whichever SNP analyzed showed a protective dominant minor allele relative risk *R*≍0.40 (*P* ≤ 4 × 10^−4^), confirming the GWAS results. The second most significant effect was the M×M interaction involving rs1508595 and rs6259 (relative risk 2.2, *P* = 0.07, with a similar result when rs995030 was analyzed). In a model restricted to the two most prominent effects, the effect of inherited genotypes in *KITLG* is maintained, and the M×M interaction is also nominally significant between rs1508595 and rs6259 (*P* = 0.03). In an analysis assuming a multiplicative model for the main effects and dominant interaction terms, the results were very similar, reflecting the low number of minor homozygotes (results not shown).

**TABLE V:** TGCT family data analysis with Equation (1)

Analysis model	Locus 1^a^	Locus 2^a^	*R* (*P-*value)^b^	*S* (*P*-value)^b^	Φ (*P*-value)^b^	ϑ (*P*-value)^b^
Full	*KITLG*/rs995030	*SHBG*/rs6259	**0.41 (3 × 10^−4^)**	1.05 (0.84)	1.34 (0.56)	1.63 (0.26)
	*KITLG*/rs1508595		**0.37 (4 × 10^−4^)**	0.85 (0.55)	1.32 (0.59)	2.19 (0.07)
Restricted to two effects	*KITLG*/rs995030	*SHBG*/rs6259	**0.43 (2 × 10^−4^)**	-	-	1.84 (0.12)
	*KITLG*/rs1508595		**0.40 (2 × 10^−4^)**	-	-	**2.19 (0.03)**

^a^Minor allele frequencies are 0.14 for rs995030, 0.17 for rs1508595, and 0.20 for rs6259.

^b^Estimated relative risks under a dominant model for locus 1 inherited genotype (*R*), locus 1 maternal genotype (*S*), two-locus M×O (Φ) and M×M (ϑ) interactions.

Significant results at *p* < 0.05 are highlighted in boldface.

## DISCUSSION

Maternal risk factors for child disease include environment exposures and genetic effects (i.e., untransmitted alleles) and it has been suggested that maternal genetic effects might contribute to the so-called “missing heritability” [Nadeau, 2009; Zhou et al., 2011]. Different designs and methods have been used to analyze maternal exposures and their interactions with offspring genotypes, including at genomewide scale [Beaty et al., 2011]. Most often, the designs and/or analysis methods do not allow for a full investigation of all the effects that can potentially be involved. Here, we revisit the log-linear approach for family trio analysis to investigate its efficiency to detect intergenerational epistasis. We propose fitting a full model in stage 1 to avoid confounding and to drop the least prominent effects in a second stage to increase the power. As with any regression analysis, if the same data are used for model selection and testing of parameters, there will be some inflation of type 1 error rate [Hurvich and Tsai, 1990], so that results must be interpreted bearing this in mind, or, preferably but often unrealistically, an independent dataset should be used. The approach can be applied to genome-wide studies but requires prioritizing the analysis by choosing which locus will be tested for both maternal and inherited genotype effects (locus 1) or only for its interactions (locus 2). We have only examined the performance of the methods under the assumption that the two interacting loci are unlinked and not in LD.

Overall findings of this study are that: (1) two-locus log-linear modeling is effective in detecting M×O and M×M interactions; (2) similar power can be achieved to detect M×O and M×M interactions of similar strengh; this power can be substantial, although lower than the power to detect the main effects; (3) M×O and M×M interactions are conditionally independent from paternal genotypes but are inflated by missing maternal genotypes; (4) in scenarios where HWE applies, its assumption increases the power to detect main effects but does not affect the interaction tests; (5) M×O and M×M tests are robust to the violation of mating symmetry assumption but, as has already been observed in single-locus models, the main maternal effect needs to be included in the model, although it is not itself robust to the violation of this assumption [Sinsheimer et al., 2003]; (6) in testing for interaction effects, the method is insensitive to any inacurracy in the second locus genotype distribution, and specifying this distribution is as powerful as estimating it in the model; (7) if the second locus genotype distribution is well specified, the method is valid to test the main effect of the second locus; (8) the risk to testicular cancer may be increased by an interaction between maternal variants in *KITLG* and *SHBG*.

The main limitation of the approach is sample size requirements, which is a common feature of all interaction analyses. However, the power to detect intergenerational interactions is reasonable and comparable to the power to detect intragenerational interactions or single-locus M×O interaction. With 300 trios, a dominant relative risk of 1.5 is detectable with 70% power in the restricted model, while power approaches 100% in samples of 1,000 trios (Fig.). The full model can be subject to multicollinearity in small datasets or when the analyzed allele is too rare. This step should be used to avoid mutual confusion of different effects if there is no collinearity, and a second analysis step modeling only the most prominent effects can increase the power, although this is subject to the above caveats.

The application of the method to TGCT confirmed the finding of two genome-wide studies: inherited *KITLG* variants (minor alleles) have a strong protective effect against the disease (relative risk 0.4, *P* = 2 × 10^−4^, assuming a dominant model). The method further detected a nominally significant M×M interaction between *KITLG* rs1508595 and *SHBG* rs6259 (reduced model relative risk = 2.2, *P* = 0.03). A simulation of similar conditions to this real dataset shows that we had 60% and 77% power to detect this interaction in full and reduced models, respectively. There was no significant main maternal effect, and a model including imprinting showed that it was not significant. TGCT has an early age of onset and a good response to chemotherapy [Rapley and Nathanson, 2010]. Its familial risk is higher than for other cancers, yet linkage studies were not successful in identifying susceptibility loci. The relative risk conferred by *KITLG* is one of the largest published so far and it has been replicated within and across studies. These unusual characteristics may derive from complex biological mechanisms such as the one reported here, although a larger sample is needed for confirmation. Both *KITLG* and *SHBG* have been previously implicated in TGCT, and if their interaction could be verified it would consolidate the observation made in in vitro studies that *KITLG* is activated by estrogen.

It has been demonstrated for single-locus analysis that power and the ability to check assumptions can be enhanced by supplementing the case-parent trios with unrelated controls and one or both parents [see e.g., Vermeulen et al., 2009], and some of these benefits are also likely to apply when two loci are under consideration. This investigation was motivated by the analysis of an existing dataset of testicular cancer cases and their parent(s). Study design is often largely driven by practical considerations; since testicular cancer primarily affects young men, recruiting testicular cancer cases attending a clinic, often accompanied by a parent, was an efficient recruitment strategy, whereas recruiting unaffected young men and their parent(s) may be more difficult. Thus, the focus of our investigation was on methods of analysis rather than design.

## Notes

Disclaimer: Supplementary materials have been peer-reviewed but not copyedited.

TABLE S1. Second locus genotype frequency estimates from 1000 simulations using Eq. 2 in models with mating type symmetry or asymmetry (median and inter-quartile range) TABLE S2. Risk parameter estimates with full and incomplete trios in dominant models (Eq. 1, full model) (median and inter-quartile range) TABLE S3. Risk parameter estimates with 1000 full trios in unconstrained analysis of datasets simulated with symmetrical parental mating (Eq. 1, full model) (median and inter-quartile range) TABLE S4. Parameter estimates with 1000 full trios in unconstrained analysis of datasets simulated under parental mating asymmetry (Eq. 1, full model) (median and inter-quartile range) TABLE S5. Parameter estimates using 1000 families with and without missing fathers in unconstrained analysis of datasets simulated under parental mating asymmetry (Eq. 1, full model) (median and inter-quartile range) TABLE S6. Estimated power and type 1 error (%) for dominant or codominant main effects of one locus in full models assuming parental mating symmetry in the analysis when symmetry or asymmetry was simulated (1000 simulations, α = 0.05) TABLE S7. Impact of misspecifying the second locus genotype frequencies on the validity to test its main effect in a full model (1000 simulations, α = 0.05) TABLE S8. Power (%) to detect the interactions at nominal 5% level using 1000 families of which some mothers are ungenotyped at both loci (1000 simulations, full model) TABLE S9. Power and type 1 error for two-locus interactions at nominal 5% level with 1000 families including missing fathers when mating asymmetry is simulated but the symmetry is assumed in the analysis (Full model) TABLE S10. Robustness to model misspecification: power and type I error of Eq. 1 assuming a dominant mode of action when codominance was simulated (full trios and some missing fathers with a total of 1000 families in 1000 simulations) TABLE S11. Risk parameter estimates under a misspecified genetic model: Analysis assuming dominant interactions when codominance was simulated (Full trios and 80% missing fathers, 1000 simulations) (median and inter-quartile range)
