# Supplementary material for: Analysis of Genetic Interactions Involving Maternal and Offspring Genotypes at Different Loci: Power Simulation and Application to Testicular Cancer
Source: Genet Epidemiol. 2012 Jun;36(6):612–21. doi: 10.1002/gepi.21655 (PMC3504980; doi:10.1002/gepi.21655)
Supplement: Supplementary file 1 — TABLE S1. Second locus genotype frequency estimates from 1000 simulations using Eq. 2 in models with mating type symmetry or asymmetry (median and inter-quartile range) TABLE S2. Risk parameter estimates with full and incomplete trios in dominant models (Eq. 1, full model) (median and inter-quartile range) TABLE S3. Risk parameter estimates with 1000 full trios in unconstrained analysis of datasets simulated with symmetrical parental mating (Eq. 1, full model) (median and inter-quartile range) TABLE S4. Parameter estimates with 1000 full trios in unconstrained analysis of datasets simulated under parental mating asymmetry (Eq. 1, full model) (median and inter-quartile range) TABLE S5. Parameter estimates using 1000 families with and without missing fathers in unconstrained analysis of datasets simulated under parental mating asymmetry (Eq. 1, full model) (median and inter-quartile range) TABLE S6. Estimated power and type 1 error (%) for dominant or codominant main effects of one locus in full models assuming parental mating symmetry in the analysis when symmetry or asymmetry was simulated (1000 simulations, α = 0.05) TABLE S7. Impact of misspecifying the second locus genotype frequencies on the validity to test its main effect in a full model (1000 simulations, α = 0.05) TABLE S8. Power (%) to detect the interactions at nominal 5% level using 1000 families of which some mothers are ungenotyped at both loci (1000 simulations, full model) TABLE S9. Power and type 1 error for two-locus interactions at nominal 5% level with 1000 families including missing fathers when mating asymmetry is simulated but the symmetry is assumed in the analysis (Full model) TABLE S10. Robustness to model misspecification: power and type I error of Eq. 1 assuming a dominant mode of action when codominance was simulated (full trios and some missing fathers with a total of 1000 families in 1000 simulations) TABLE S11. Risk parameter estimates under a misspecified genetic model: Analysis assum [file gepi0036-0612-sd1.pdf]

Analysis of Genetic Interactions Involving Maternal and Offspring Genotypes at Different Loci: Power Simulation and Application to Testicular Cancer

Journal:	*Genetic Epidemiology*
Manuscript ID:	GenEpi-11-0216.R3
Wiley - Manuscript type:	Research Article
Date Submitted by the Author:	10-May-2012
Complete List of Authors:	Nsengimana, Jeremie; University of Leeds, Section of Epidemiology and Biostatistics, Leeds Institute of Molecular Medicine<br>Barrett, Jennifer; University of Leeds, Section of Epidemiology and Biostatistics, Leeds Institute of Molecular Medicine
Key Words:	Family trios, Two-locus log-linear model, EM algorithm, Type-1 error

SCHOLARONE™
Manuscripts

We are pleased that the reviewers now feel we have addressed most of their concerns. Below are our responses to the new comments.

Reviewer: 1
Comments to Author
These revisions are clear and well described.

I only have a comment about the new equations 1 and 2 which need not affect the final version: the authors recognise that the main effect and genotype frequency cannot both be identified, and offer two approaches: equation 1 assumes the genotype frequency is known, allowing estimation of the main effect when the former is correctly specified; equation 2 estimates a confounded combination of the two parameters. It should be clear that the two equations lead to identical estimates of all other parameters and are therefore equivalent for inference of the interaction effects. This is borne out by table 4 in which all results for eqns 1 and 2 are identical, except for simulation 8 (is this a typo?). This might not be clear to some readers since eqn 2 is billed as an "alternative approach" and substantial comparisons are made between the two equations even when they are equivalent by construction.

We have clarified in the manuscript that equations 1 and 2 represent essentially the same analysis method, the only difference being in the treatment of genotype frequencies. However we think that it is informative for the reader to know that the two options are possible, i.e. fixing genotype frequencies or estimating them (confounded with main effects) within the model. All results concerning the interaction parameters from these two analysis options are the same, which is to be expected as the reviewer points out. (The difference for model 8 was a typo. Thank you for highlighting this).

Reviewer: 2
Comments to Author
The authors have improved the manuscript. My only remaining major criticism concerns the use of dominant models and some confusion in the manuscript as to when they are assuming dominant effects in the analysis and when they are not:
(1) The authors simulate data using both dominant and multiplicative effects. They state that they are analyzing the data under the full model and then refer the reader to equation (1) where the genetic effects can be codominant. However, in the legend to table II they state that the data are analyzed assuming dominant effects. The use of the term full model and referring to equation (1) led me to believe that they were allowing for co-dominant (genotypic) models in the results shown (e.g. table IV). The authors should be more explicit about what they mean by the full model, I believe they mean that they are estimating maternal and child main effects along with the interactions but they could be putting genetic model restrictions on those models but that is not clear. They should also be very clear in the text when they are assuming particular genetic effects in their analyses.

We are sorry for the confusion caused by the use of certain terms. We have clarified in the Methods section that we call “full model” a model that includes MxM and MxO interactions along with the main effects, irrespective of the actual genetic mode of action, and we indicated for each set of results which model was used (full or reduced) and which genetic mode of inheritance was assumed (dominant or codominant).

(2) A major reason for the simulations presented by the authors is so they can be certain that they are analyzing the TGCT data with an appropriate model. In this analysis they assume dominant effects based on their marginal analyses using cases and controls I believe. What would be the consequences on their analyses if the minor alleles are not acting in a dominant manner? The authors can easily examine the question of genetic model misspecification by extending

For Peer Review

the simulations. They should take the simulations that they performed under the multiplicative genetic model and analyze them under a dominant model to determine the effect.

In our reply to reviewer 2 in the first revision (comment 6B), we highlighted that the method we are proposing can test up to 12 parameters while also fitting a large number of nuisance parameters. We explained that it would be impractical to apply such a “saturated” model to a small dataset and that is why we set up simulations under a dominant mode of action since we intended to evaluate the method under scenarios of small and moderate sample sizes. We have now applied this dominant model to the datasets simulated under a multiplicative model and we show that the test for interaction remains valid with similar power (Tables S10 and S11). The only negative aspect of model misspecification is the inaccuracy of parameter estimation, which is inevitable. In fact, from the published case-control analyses a multiplicative model fits the TGCT data well (heterozygote and major homozygote OR=2.6 and 6.7 respectively in the replication set).However, with a minor allele frequency of 0.14, we expect only 2% homozygote frequency, so in this small data set we chose to combine these genotypes in a dominant model. We have also carried out an analysis assuming multiplicative main effects along with dominant MxM and MxO interactions, and all results were very similar. We have added further details on this to the manuscript.

Minor criticism:
Although the authors have improved the discussion of problems estimating the main effects of the second maternal locus, I believe that potential bias in the estimate of these effects and inference concerning them should be made abundantly clear by adding a sentence like "It is important to note that this second locus main effect is biased and inference regarding its existence is not accurate" in the results section - page 12 line 45.

Table S7 shows that the second locus main effect can be only accurately estimated when its genotype frequency distribution is correctly specified. This table is commented on page 14, and we have now further emphasised this on page 12 as suggested.

For Peer Review

Analysis of Genetic Interactions Involving Maternal and Offspring Genotypes at Different Loci: Power Simulation and Application to Testicular Cancer

Jérémie Nsengimana[1*] and Jennifer H Barrett[1]

[1] Section of Epidemiology and Biostatistics, Leeds Institute of Molecular Medicine, University of Leeds, Leeds, UK

[*] **Communication to:**

Jérémie Nsengimana

Section of Epidemiology and Biostatistics

Leeds Institute of Molecular Medicine

Saint James Hospital

Cancer Genetics Building

Beckett Street

Leeds LS9 7TF

United Kingdom

Email: J.Nsengimana@leeds.ac.uk

Phone: +44 1132066531

Fax: +44 1132340183

Running title: Maternal-offspring two-locus interactions

For Peer Review

ABSTRACT

The analyses of genetic interaction between maternal and offspring genotypes are usually conducted considering a single locus. Here we propose testing maternal x offspring (MxO) and maternal x maternal (MxM) genotype interactions involving two unlinked loci. We reformulate the log-linear approach of analyzing cases and their parents (family trios) to accommodate two loci, fit fuller models to avoid confounding in a first analysis step and propose that the model be reduced to the most prominent effects in a second step. We conduct extensive simulations to assess the validity and power of this approach under various model assumptions. We show that the approach is valid and has good power to detect MxO and MxM interactions. For example, the power to detect a dominant interaction relative risk of 1.5 (both MxO and MxM) is 70% with 300 trios and approaches 100% with 1000 trios. Unlike the main effects, MxO and MxM interactions are conditionally independent of mating types, and consequently their power is not affected by missing paternal genotypes. When applied to single locus MxO interaction, our method is as powerful as other existing methods. Applying the method to testicular cancer, we found a nominally significant MxM interaction between SNPs from C-Kit Ligand (*KITLG*) and Sex Hormone Binding Globulin (*SHBG*) using 210 families (relative risk 2.2, p=0.03). This finding supports a role of maternal hormones in offspring testicular cancer and warrants confirmation in a larger dataset.

Key words: Family trios, EM algorithm, two-locus log-linear model, type-1 error

For Peer Review

INTRODUCTION

It has been suggested that maternal effects play an important role in species adaptation [Wolf 2000], and there is a compelling body of evidence that complex adulthood diseases such as diabetes, hypertension, schizophrenia, asthma, testicular, renal and gastric cancers are partially determined by antenatal factors [Drever et al., 2010; Eriksson et al., 2011; Maringhini et al., 2010; Winder et al., 2011]. Although the process appears to be dominated by *in-utero* epigenetic changes, placental size and foetal growth rate, all of which are directly related to maternal diet [Barker et al., 2010], maternal genes involved in placental formation, adhesion, vascularisation, hormone secretion or transport activity are also likely to be involved [Jansson et al., 2002].

Dissecting maternal effects is attracting renewed attention, and novel statistical methods and designs, such as those exploiting Assisted Reproduction Technology (ART), are emerging [Rice et al., 2009; Thapar et al., 2007; Zhou et al., 2011]. ART allows complete separation of the effects of the child's genotype from those of the maternal genotype when gestational and biological mothers are different. More classical family designs have also been used to jointly analyse these effects [Weinberg et al., 1998], with the possibility to include maternal-foetal interaction at one locus [Ainsworth et al., 2011; Sinsheimer et al., 2003]. Effects that are seldom analysed are the interaction between a maternal genotype at one locus and a child genotype at another locus, and the interaction of two maternal loci to raise the disease risk in their offspring. Maternal-offspring (MxO) interaction at different loci is ubiquitous in mammals [Wolf, 2000]. In a mouse model, it was shown that maternal thyroid hormones bind the embryonic thyroid receptors [Nucera et al., 2010] and a list of 81 foetal genes have been identified that are maternally regulated [SenthamaraiKannan et al., 2011]. MxO interactions may partially determine birth-weight, a trait associated with many adulthood diseases [Wolf, 2000]. Simulation

For Peer Review

studies have shown that when MxO interaction exists, the maternally expressed allele may persist longer at a higher frequency in the population than the zygotically expressed allele, and under certain genetic models, it escapes from the purifying selection, increasing the disease prevalence in the population [Priest and Wade, 2010]. An interaction between two maternal genes (MxM interaction) can also increase the child disease risk. For example an association was found between craniofacial malformations and an interaction between maternal single nucleotide polymorphism (SNP) genotypes in folate and choline metabolism genes [Mostowska et al., 2010a; Mostowska et al., 2010b].

The risk of developing testicular germ cell tumour (TGCT) has long been associated with antenatal exposure to maternal hormones. When exposed to high levels of estrogens *in vitro*, primordial germ cells (PGCs, the embryonic precursors of gametes) start transcription of C-kit ligand (*KITLG*), and the accumulation of this growth factor eventually transforms PGCs into tumorigenic cells [Moe-Behrens et al., 2003]. We and others have found that SNPs from *KITLG* are the most associated with TGCT in genome-wide association studies (GWAS) [Kanetsky et al., 2009; Rapley et al., 2009] and it can be hypothesised that the disease-initiating events occur in the early embryonic development from an interplay between maternal estrogens and child or maternal *KITLG*. The purpose of this study is to investigate the application of log-linear methods to test the interactions between a maternal genotype at one locus and a child genotype at another unlinked locus and between two unlinked maternal loci. We use a method originally proposed to analyse gene-environment interactions [Umbach and Weinberg, 2000], considering as exposure a second locus maternal genotype. Our study extends and investigates this approach in several ways: Firstly, because genetic effects from maternal and child genotypes can be mutually confounded we fit a model including the main effects from child and maternal genotypes along

For Peer Review

with two-locus MxO and MxM interactions. We propose that a further analysis stage may be conducted, where the least significant effects are dropped from the model and we discuss the implications of this model selection process. Secondly, we simulated data to assess the relative merits (power and type-1 error) of different approaches to testing for MxO and MxM interactions: testing assuming mating symmetry, either specifying or estimating the second locus genotype frequency distribution, and testing assuming Hardy-Weinberg equilibrium (HWE). Thirdly, we investigate the sensitivity of the MxO and MxM interactions test to the violation of the assumption of parental mating symmetry. Fourthly, we compare the power to detect the interactions to the power to detect main effects. Finally, we use the EM algorithm to analyse incomplete trios and show that MxO and MxM interaction tests are independent of missing paternal genotypes. Therefore, although the methods considered in this study have been used previously, we revisit them from a different angle, reformulating the log-linear framework to accommodate two-locus models and exploring various novel aspects before conducting the power simulations. We then apply the method to a TGCT dataset, examining the interaction between a maternal hormone-related gene and *KITLG*.

For Peer Review

METHODS

TWO-LOCUS LOG-LINEAR MODEL

We assume that family trios have been genotyped at a first locus and that the mothers are additionally genotyped at a second locus, both unlinked and in linkage equilibrium with the first locus. Although some of the tests we propose rely only on the assumption of linkage equilibrium, MxO and MxM cannot be tested simultaneously without imposing the absence of both linkage and linkage disequilibrium (LD). Therefore, throughout this article when we say “unlinked” we also imply absence of LD. Originally, the log-linear method was designed to analyse genetic

effects from one locus in cases, their mothers, or both, and possibly subject to imprinting [Weinberg et al., 1998]. Under the assumption of mating symmetry, joint genotypes of parents define 6 mating types, and joint genotypes of a family trio define 15 categories (see Table I). If mothers of cases are additionally genotyped at a second locus (M_2), the 15 categories can be stratified on the second locus genotypes and the proportions of family trios falling in each of the new defined categories is given in Table I. Using the original notation of Weinberg et al. [1998], locus 1 has main maternal effects S_i and main offspring effects R_j, where *i, j* represent the number of susceptibility alleles carried by mother and child respectively. S_i and R_j are the relative excess of disease risk in the offspring conferred by maternal and offspring disease alleles. Scaling factors μ_m are population frequencies of the mating types.

For Peer Review

We introduce two new sets of parameters: ϑ_{ik} coefficient for MxM interaction between the two loci and Φ_{jk} for MxO interaction (index k is the number of maternal alleles at locus 2, assumed to be unlinked to locus 1). Stratifying the expected cell counts by the second locus implies that each of the new cells must be weighted by the population genotype frequencies p_k at this locus. Using Table I, a two-locus log-linear model of family trio counts can be written as:

$$\ln\left(n_{M=i,F=f,C=j,M2=k}\right) \propto \ln\left(\mu_m\right) + \delta_k + \alpha_i + \beta_j + \varphi_{ik} + \phi_{jk} + \ln[p_k 2^{I_{i=j=f=1}}] \qquad \text{(Eq.1)}$$

where $n_{M=i,F=f,C=j,M2=k}$ is the expected number of family trios with specific maternal (M), paternal (F) and child (C) genotype at locus 1 and maternal (M_2) genotype at locus 2. Mating type index m on the right-hand-side of the equation ranges from 1 to 6 as (M,F) on the left-hand-side takes the values (2,2), (2,1), (2,0), (1,1), (1,0) and (0,0). Regression coefficients α_i, β_j, φ_{ik} and ϕ_{jk} are

respectively the natural logarithm of disease relative risks S_i, R_j, ϑ_{ik} and Φ_{jk} of Table I. All these coefficients are set to zero for the baseline genotype, i.e. $\alpha_0=\beta_0=0$, $\varphi_{ik}=0$ if $i=0$ or $k=0$, and $\phi_{jk}=0$ if $j=0$ or $k=0$. The last term of the equation represents a "weight" or "offset". There are 2 different ways giving rise to a heterozygous offspring when both parents are heterozygous, and they are equally likely in the absence of imprinting. In this case the cell count is weighted by a factor of 2, which is reflected in the "offset" (i.e. the indicator variable $I_{i=j=f=1}$ takes the value of 1). In addition, with a second locus, each cell count is proportional to the genotype frequencies p_k as shown in Table I, and this is also reflected in the "offset" term.

Parameter δ_k is the natural logarithm of τ_k shown in Table I, which fits the main effect of maternal genotype k at locus 2, assuming the absence of effect from the baseline genotype ($\delta_0=0$). Although we are not interested in this effect, its inclusion in the model is necessary to ensure that the interaction test does not depend on genotype frequencies. On closer inspection, it can be seen that each of the new cells created by stratifying on locus 2 is weighted by genotype frequencies independently of coefficients ϑ_{ik} and Φ_{jk} (Table I). It follows that if one were interested in testing the locus 2 main effect, knowing its frequency distribution with precision would be required. It would also be required if one fitted the interaction terms without this main effect in the model. However, as we will show in simulations, estimates of the interaction terms are independent of the genotype frequencies provided that the main effect is included. Furthermore, Eq.1 is valid for testing the main effect δ_k itself when the genotype frequencies are correctly specified in the offset term (see Results).

For Peer Review

Eq.1 requires specifying the second locus genotype frequencies in a similar way to the environmental risk factor ratios used by Umbach and Weinberg [2000] (see their equation on page 257). However, as we consider a three-level exposure (second locus genotype) we fit a two-level main effect (δ_k, k=1,2) instead of one level. Their term ϖ, which is the natural logarithm of the ratio of exposed to unexposed individuals in the population, would be written as $\ln(p_k/p_0)$ in our notation. The Umbach and Weinberg [2000] term μ_i is then equivalent to $\ln(\mu_m)+\ln(p_0)$ in our notation.

The locus 2 genotype frequencies could be estimated, instead of specifying them in an offset term as in Eq.1. However, now the second locus main effect cannot be estimated. Eq.2 describes this model:

$$\ln\left(n_{M=i,F=f,C=j,M2=k}\right) \propto \ln\left(\mu_m\right) + \lambda_k + \alpha_i + \beta_j + \varphi_{ik} + \phi_{jk} + \ln(2^{I_{i=j=f=1}}) \qquad \text{(Eq.2)}$$

where λ_k equals $\ln(p_k/p_0)$, k=1,2. Using the ratio of frequencies in the model ensures that their sum is constrained to equal to 1.

An alternative approach to reduce the number of parameters is to use Eq.1 and assume HWE for both loci. In fact, μ_m can be expressed as a function of one allele frequency if HWE is assumed at locus 1 [Weinberg et al., 1998]. However, unlike the main effects S_i and R_j that depend directly on the mating types, estimates of the interaction terms ϑ_{ik} and Φ_{jk} are conditionally independent of these nuisance parameters when S_i and R_j are fitted in the model. To see this, note that in Table I the interaction coefficients ϑ_{ik} and Φ_{jk} are defined in columns where M_2=1 and M_2=2 and they are tested against the baseline column M_2=0. They are independent from mating type strata because the same coefficient μ_m multiplies cell counts in these 3 columns on each row of Table I.

For Peer Review

Therefore, modelling all mating types or replacing them by one parameter does not affect the power to detect the interactions (See Results).

Using Eq.1 or Eq.2, MxM and MxO interactions can be tested in a likelihood ratio test of the full model including all the effects *versus* a model excluding the effect investigated. Log-linear methods also allow the analysis of MxO interactions involving one locus (coefficient ρ_{ij} in Eq.3, defined for i>0 and j>0, set to zero when if i=0 or j=0). A likelihood ratio test of the interaction term ρ_{ij} is equivalent to the multinomial test used in the EMIM software [Ainsworth et al., 2011] and to the method of Sinsheimer et al. [2003] implemented in the software package Mendel [Lange et al., 2001].

$$\ln\left(n_{M=i,F=f,C=j}\right) \propto \ln\left(\mu_m\right) + \alpha_i + \beta_j + \rho_{ij} + \ln(2^{I_{i=j=f=1}}) \qquad \text{(Eq.3)}$$

INCORPORATING INCOMPLETELY GENOTYPED TRIOS

The EM (Expectation-maximisation) algorithm is a powerful approach to jointly analysing full trios and incompletely genotyped trios in the log-linear framework [Weinberg, 1999]. For the analysis of MxM and MxO interactions, tests from Eq.1 and Eq.2 are completely immune to missing paternal genotypes. This is because, as said earlier, the interaction terms ϑ_{ik} and Φ_{jk} are conditionally independent of the mating type strata when S_i and R_j are included in the model. In other words, missing fathers cause an ambiguity of mating types which could have an impact on the power to detect main effects S_i and R_j, but the interactions ϑ_{ik} and Φ_{jk} are completely unaffected. However, when the missing parent is the mother, it is reasonable to assume that her genotypes will be unavailable for both loci, in which case the data contain little information about

For Peer Review

locus 2. We investigate the method's efficiency for MxM and MxO interactions when the EM algorithm is used to analyse trios with missing parental genotypes. More information on the application of the EM algorithm to incomplete family trios can be found in Weinberg [1999].

SIMULATIONS

We designed simulations to evaluate the type-1 error and power of Eq.1 and Eq.2 to test two-locus MxM and MxO interactions as well as Eq.3 for single locus MxO interaction. In all simulations, we considered one locus genotyped in family trios (locus 1) and another, unlinked and in linkage equilibrium in the general population, genotyped only in the mothers (locus 2). In all simulated models, 1000 replicates were generated where the baseline disease incidence rate was 0.1, for both loci HWE was assumed and the risk allele frequency (RAF) was 0.3. Under dominant inheritance (Table II), we simulated one example of a small dataset (300 family trios) and one moderate sample size (1000 family trios). Only the sample size of 1000 trios was considered for codominant genetic inheritance (Table III). Some of the simulated models were intended to assess type-1 error or power for both MxM and MxO interactions, while other models were designed to assess the type-1 error for one type of interaction and the power for the other. These models therefore allow possible confounding between the two effects to be assessed. They also allow an evaluation of the power gain in models restricted to only the genuine effects compared to a more saturated model. Models 11a-14a (Table III) were simulated under parental mating asymmetry to assess the sensitivity of the interaction tests to violation of the mating symmetry assumption. In these models, locus 1 RAF was 0.3 in females, but two equally sized strata of males were considered: one with RAF 0.1 and another with RAF 0.5, with HWE within each stratum. This corresponds to an overall RAF of 0.3 for males in the combined population, but gives rise to asymmetry in the mating patterns, since, for example, a female is now more

For Peer Review

likely to be heterozygote than is a male. For locus 2, the RAF remained 0.30 in females and in each of the two male strata, and the 2 loci were unlinked as before.

To demonstrate the independence of interaction tests from genotype frequency specification, datasets simulated under model 1 (see Table II) were analysed assuming a broad range of the locus 2 genotype frequency distributions. To illustrate the immunity of the interaction tests to missing paternal data, we generated missing paternal genotypes in 10-80% of families in model 1 simulations. To assess the ability of the EM algorithm to handle missing data for two loci, we simulated missing maternal genotypes in 5-30% of families. Using data simulated under parental mating symmetry, we further compare (1) the power of detecting two-locus MxM and MxO interactions using Eq.1 and Eq.2, both assuming mating symmetry and Eq.1 assuming HWE (Eq.1_hwe), (2) the power to detect these interactions *vs.* the power to detect the main effects S_i and R_j of similar magnitude and (3) the power to detect single locus MxO interaction using Eq.3 *vs.* EMIM. The analysis including the main child and maternal effects from locus 1, a main effect from locus 2 and the two-locus MxO and MxM interactions under dominance or codominance will be referred to as a full model, while the analysis restricted to a subset of these effects will be referred to as a reduced or restricted model. Although our primary focus is on the validity and power of the interactions tests we also report as supplementary data the risk parameter estimates for the simulated models. All data were simulated with a purpose-built C program and analysed in STATA using Poisson regression. These programs are available from the authors on request.

APPLICATION TO TGCT

We applied two-locus log-linear models to 210 families (147 full trios and 63 case-mother pairs), testing the interactions between *KITLG* SNPs (rs1508595 and rs995030, each separately) and

For Peer Review

rs6259, a missense SNP from *SHBG* (Sex Hormone Binding Globulin; it binds estrogens and other sex hormones, regulating their availability). The 2 *KITLG* SNPs were the most significant in a TGCT GWAS and showed independent signals in multivariable analysis (Rapley et al., 2009). The linkage disequilibrium (LD) between *KITLG* SNPs was estimated using 640 subjects (370 case parents and 270 unrelated controls) with STATA add-on command *pwld*.

RESULTS

VALIDITY AND POWER OF TWO-LOCUS INTERACTION TESTS IN FULL TRIOS

Table IV summarises the type-1 error and power to detect MxM and MxO interactions at the nominal significance level of 0.05 in different simulated datasets with 300 or 1000 trios. There are 5 sections in the table: the top section shows for simulated models 1 and 6 the type-1 error of the interactions using Eq.1 or Eq.2, both assuming mating symmetry. For model 1, this section further depicts the robustness of Eq.1 to misspecifying the second locus genotype frequencies and assesses the type-1 error of Eq.1 under the assumption of HWE at locus 1 (Eq.1_hwe). All the results in this section show that the interaction tests have a non-inflated type-1 error using all the methods. The highest error observed is 5.8%, which is not significantly higher than 5% (one-sided p-value for the difference is 0.14). Results also confirm that the interaction tests based on Eq.1 are robust to genotype frequency misspecification: data analysis assuming a frequency distribution of 0.01, 0.18, 0.81 as well as 0.25, 0.50, 0.25 gave exactly the same interaction error rate as when the correct genotype frequencies of 0.09, 0.42 and 0.49 were used. As explained earlier, this is because the uncertainty is compensated for by the inclusion of the second locus main effect. However, it is important to note that this main effect can only be accurately estimated if the genotype frequency distribution is correctly specified (see next paragraph). Model 6 results show that main and interaction effects are not confounded if they are all fitted.

For Peer Review

The second section of Table IV shows models 2-5 where one type of interaction was simulated under the null while the other was under the alternative hypothesis. The results in this section show that the interaction tests are always valid and have reasonable power. For example, in simulation model 3 with 300 trios, the MxO test has a type-1 error rate of 4.5% and the MxM test has a power of 73.6% at a nominal 5% significance level. The third section of Table IV shows the power for both MxM and MxO interactions (model 7 and 8) using Eq.1, Eq.1_hwe and Eq.2. In all simulated models and sample sizes, Eq.1 and Eq.2 have the same power to detect the interactions, since the only difference between them is in the treatment of the confounded main effects and genotype frequencies, and Eq.1_hwe shows similar power. For example, in model 8 with 300 families, the powers are 65.0% and 66.6% for the two interactions with Eq.1 and Eq.2 while they are 65.9% and 67.5% with Eq.1_hwe. Results in the fourth section of Table IV (models 11 to 14) were obtained assuming an unconstrained genotypic model estimating 12 different effects and 8 nuisance parameters. They show that MxO and MxM tests remain valid and retain good power with 1000 full trios. Results in the last section of Table IV show that two-locus interaction tests remain valid and do not lose power when true parental mating is asymmetric but symmetry is assumed in the analysis.

Supplementary Table S1 shows that the frequencies of the second locus genotypes are accurately estimated with Eq.2, although this is based on the assumption that the second locus has no main effect. Risk parameter estimates using Eq.1 are unbiased and generally close to their true values (Tables S2-S5). These risk estimates remain accurate when parental mating asymmetry is simulated but symmetry is assumed in the analysis.

For Peer Review

POWER FOR MAIN EFFECTS AND SINGLE LOCUS MxO INTERACTION

Although assuming HWE does not improve the power to detect MxM and MxO interactions (see Table IV), it does improve the power for the main effects as shown in supplementary Table S6 (model 6). Overall, the power to detect the main effects is substantially higher than the power to detect interactions. As has been reported by others [Sinsheimer et al., 2003], we found that assuming mating type symmetry in testing the main child effect remains valid when the mating is asymmetric while testing the main maternal effect is invalid (Table S6, model 13a). We stated in the Methods section that Eq.1 could be used to test the second locus main effect when its genotype frequency distribution is known, and Table S7 gives a confirmation of this statement. However, a small deviation from the correct frequency can cause considerable effect inflation, invalidating the test of the main effect. Using the data simulated in model 1, we fitted Eq.3 to assess its type-1 error for single locus MxO interaction. The type-1 error found at level 0.05 was 5.2% with 1000 trios and 5.5% with 300 trios. With sample sizes 300 and 1000 trios we found that Eq.3 and EMIM have similar power to detect single locus MxO interactions (Fig.1).

MODEL REDUCTION

All the results presented so far were obtained when fitting a full model. While this full model is necessary to detect all the existing effects, the large number of terms impacts on the power to detect each of them. Restricting the analysis to the truly simulated effects (in addition to locus 2 main effect terms when there is an interaction involving this locus), the power is substantially increased, as can be seen in Fig. 1.

For Peer Review

TYPE-1 ERROR AND POWER FOR THE INTERACTIONS WITH MISSING PARENTS

The validity, power and estimation accuracy of the MxM and MxO interaction tests using Eq.1 are not affected by missing fathers up to 80% (Fig. 2, Fig.3 and table S2). By contrast, when genotypes are missing in mothers of cases at the two loci considered, both interaction tests have an inflated type-1 error. In an initial sample size of 300 trios, there is a significant inflation with 10% missing mothers or more, while this occurs with 15% or more missing mothers in an initial sample size of 1000 trios (Fig.2). With a total sample size of 1000 families, power to detect MxM and MxO interactions is unaffected by up to 10% missing mothers (Table S8) while their risk parameter estimates remain accurate with up to 20% missing mothers (Table S2). When parental mating asymmetry is simulated but the symmetry is assumed in the analysis, as much as 80% missing fathers do not affect the validity, the power or the estimation accuracy of the two-locus interactions test (Tables S5 and S9).

ROBUSTNESS TO MODEL MISSPECIFICATION

When the correct model for the interactions is codominant, assuming a dominant model in the analysis does not invalidate the test, nor does it cause a power loss (Table S10). However, as expected, the parameters estimation becomes inaccurate when the effects exist (Table S11).

TGCT DATASET ANALYSIS

Despite showing independent effects on TGCT in GWAS, we found that *KITLG* rs1508595 and rs995030 are in strong LD (D'=0.91, r^2=0.65, MAF=0.17 and 0.14 respectively). In the original results from the GWAS (Kanetsky et al., 2009; Rapley et al., 2009), where major rather than minor alleles were tested, allelic relative risks were estimated to be 3.1 and 2.6 respectively and the pattern of risk was consistent with a multiplicative genetic model. In view of the small sample

size and rarity of the minor homozygote genotype, we here carry out a primary analysis of the 147 full trios and 63 case-mother pairs assuming dominant main effects and interactions using Eq.1 in full and reduced models. Case-father pairs were not used since simulations showed that they could inflate type-1 error. The reduced model included the 2 most prominent effects in the full model. Table V reports the results: in the full model, the most significant effect was the inherited *KITLG* genotypes. Whichever SNP analysed showed a protective dominant minor allele relative risk R≈0.40 ($p \leq 4 \times 10^{-4}$), confirming the GWAS results. The second most significant effect was the MxM interaction involving rs1508595 and rs6259 (relative risk 2.2, p=0.07, with a similar result when rs995030 was analysed). In a model restricted to the two most prominent effects, the effect of inherited genotypes in *KITLG* is maintained, and the MxM interaction is also nominally significant between rs1508595 and rs6259 (p=0.03). In an analysis assuming a multiplicative model for the main effects and dominant interaction terms, the results were very similar, reflecting the low number of minor homozygotes (results not shown).

DISCUSSION

Maternal risk factors for child disease include environment exposures and genetic effects (i.e. untransmitted alleles) and it has been suggested that maternal genetic effects might contribute to the so-called "missing heritability" [Nadeau, 2009; Zhou et al., 2011]. Different designs and methods have been used to analyse maternal exposures and their interactions with offspring genotypes, including at genomewide scale [Beaty et al., 2011]. Most often, the designs and/or analysis methods do not allow for a full investigation of all the effects that can potentially be involved. Here we revisit the log-linear approach for family trio analysis to investigage its efficiency to detect intergenerational epistasis. We propose fitting a full model

in stage 1 to avoid confounding and to drop the least prominent effects in a second stage to increase the power. As with any regression analysis, if the same data are used for model selection and testing of parameters, there will be some inflation of type 1 error rate [Hurvich and Tsai, 1990], so that results must be interpreted bearing this in mind, or, preferably but often unrealistically, an independent data set should be used. The approach can be applied to genome-wide studies but requires prioritising the analysis by choosing which locus will be tested for both maternal and inherited genotype effects (locus 1) or only for its interactions (locus 2). We have only examined the performance of the methods under the assumption that the 2 interacting loci are unlinked and not in LD.

Overall findings of this study are that: (1) Two-locus log-linear modelling is effective in detecting MxO and MxM interactions, (2) Similar power can be achieved to detect MxO and MxM interactions of similar strengh; this power can be substantial although lower than the power to detect the main effects; (3) MxO and MxM interactions are conditionally independent from paternal genotypes but are inflated by missing maternal genotypes; (4) In scenarios where HWE applies, its assumption increases the power to detect main effects but does not affect the interaction tests; (5) MxO and MxM tests are robust to the violation of mating symmetry assumption but, as has already been observed in single locus models, the main maternal effect needs to be included in the model although it is not itself robust to the violation of this assumption [Sinsheimer et al., 2003]; (6) In testing for interaction effects, the method is insensitive to any inacurracy in the second locus genotype distribution, and specifying this distribution is as powerful as estimating it in the model; (7) If the second locus genotype distribution is well specified, the method is valid to test the main effect of the second locus; (8) The risk to testicular cancer may be increased by an interaction between maternal variants in *KITLG* and *SHBG*.

For Peer Review

The main limitation of the approach is sample size requirements, which is a common feature of all interaction analyses. However, the power to detect intergenerational interactions is reasonable and comparable to the power to detect intragenerational interactions or single locus MxO interaction. With 300 trios, a dominant relative risk of 1.5 is detectable with 70% power in the restricted model, while power approaches 100% in samples of 1000 trios (Fig. 1). The full model can be subject to multicollinearity in small datasets or when the analysed allele is too rare. This step should be used to avoid mutual confusion of different effects if there is no collinearity, and a second analysis step modelling only the most prominent effects can increase the power, although this is subject to the above caveats.

The application of the method to TGCT confirmed the finding of two genome-wide studies: inherited *KITLG* variants (minor alleles) have a strong protective effect against the disease (relative risk 0.4, p=2x10^{-4}, assuming a dominant model). The method further detected a nominally significant MxM interaction between *KITLG* rs1508595 and *SHBG* rs6259 (reduced model relative risk=2.2, p=0.03). A simulation of similar conditions to this real dataset shows that we had 60% and 77% power to detect this interaction in full and reduced models respectively. There was no significant main maternal effect, and a model including imprinting showed that it was not significant. TGCT has an early age of onset and a good response to chemotherapy [Rapley and Nathanson, 2010]. Its familial risk is higher than for other cancers, yet linkage studies were not successful in identifying susceptibility loci. The relative risk conferred by *KITLG* is one of the largest published so far and it has been replicated within and across studies. These unusual characteristics may derive from complex biological mechanisms such as the one reported here, although a larger sample is needed for confirmation. Both *KITLG* and *SHBG* have

For Peer Review

been previously implicated in TGCT, and if their interaction could be verified it would consolidate the observation made in *in vitro* studies that *KITLG* is activated by oestrogen.

It has been demonstrated for single locus analysis that power and the ability to check assumptions can be enhanced by supplementing the case-parent trios with unrelated controls and one or both parents [see e.g. Vermeulen, et al. 2009], and some of these benefits are also likely to apply when two loci are under consideration. This investigation was motivated by the analysis of an existing data set of testicular cancer cases and their parent(s). Study design is often largely driven by practical considerations; since testicular cancer primarily affects young men, recruiting testicular cancer cases attending a clinic, often accompanied by a parent, was an efficient recruitment strategy, whereas recruiting unaffected young men and their parent(s) may be more difficult. Thus the focus of our investigation was on methods of analysis rather than design.

For Peer Review

ACKNOWLEDGEMENTS

This study was founded by Cancer Research UK grant C588/A4994. We thank Helen Snowden, Mark Harland and Juliette Randerson-Moor for genotyping SNPs in candidate genes to support our method evaluation. We are grateful to the manuscript reviewers for their insightful comments and suggestions. We declare no conflict of interest.

REFERENCES

Ainsworth HE, Unwin J, Jamison DL, Cordell HJ. 2011. Investigation of Maternal Effects, Maternal-Fetal Interactions and Parent-of-Origin Effects (Imprinting), Using Mothers and Their Offspring. Genet Epidemiol 35(1):19-45.

Barker DJP, Gelow J, Thornburg K, Osmond C, Kajantie E, Eriksson JG. 2010. The early origins of chronic heart failure: impaired placental growth and initiation of insulin resistance in childhood. Euro J Heart Fail 12(8):819-825.

Beaty TH, Ruczinski I, Murray JC, Marazita ML, Munger RG, Hetmanski JB, Murray T, Redett RJ, Fallin MD, Liang KY and others. 2011. Evidence for Gene-Environment Interaction in a Genome Wide Study of Nonsyndromic Cleft Palate. Genet Epidemiol 35:469-78.

Drever N, Saade GR, Bytautiene E. 2010. Fetal Programming: Early-life Modulations that Affect Adult Outcomes. Current Allergy and Asthma Reports 10(6):453-459.

Eriksson JG, Kajantie E, Thornburg KL, Osmond C, Barker DJ. 2011. Mother's body size and placental size predict coronary heart disease in men. Euro Heart J 32(18):2297-2303.

Hurvich CM, Tsai CL. 1990. The Impact of Model Selection on Inference in Linear-Regression. American Statistician 44(3):214-217.

Jansson T, Ekstrand Y, Bjorn C, Wennergren M, Powell TL. 2002. Alterations in the activity of placental amino acid transporters in pregnancies complicated by diabetes. Diabetes 51(7):2214-2219.

Kanetsky PA, Mitra N, Vardhanabhuti S, Li MY, Vaughn DJ, Letrero R, Ciosek SL, Doody DR, Smith LM, Weaver J and others. 2009. Common variation in KITLG and at 5q31.3 predisposes to testicular germ cell cancer. Nat Genet 41(7):811-815.

Lange K, Cantor R, Horvath S, Perola M, Sabatti C, Sinsheimer J, Sobel E. 2001. Mendel version 4.0: a complete package for the exact genetic analysis of discrete traits in pedigree and population data sets. Am J Hum Genet 69(4):504.

Maringhini S, Corrado C, Maringhini G, Cusumano R, Azzolina V, Leone F. 2010. Early origin of adult renal disease. Journal of Maternal-Fetal & Neonatal Medicine 23:84-86.

For Peer Review

Moe-Behrens GHG, Klinger FG, Eskild W, Grotmol T, Haugen TB, De Felici M. 2003. Akt/PTEN signaling mediates estrogen-dependent proliferation of primordial germ cells in vitro. Mol Endocrinol 17(12):2630-2638.

Mostowska A, Hozyasz KK, Biedziak B, Misiak J, Jagodzinski PP. 2010a. Polymorphisms located in the region containing BHMT and BHMT2 genes as maternal protective factors for orofacial clefts. Euro J Oral Sci 118(4):325-332.

Mostowska A, Hozyasz KK, Wojcicki P, Dziegelewska M, Jagodzinski PP. 2010b. Associations of folate and choline metabolism gene polymorphisms with orofacial clefts. J Med Genet 47(12):809-815.

Nadeau JH. 2009. Transgenerational genetic effects on phenotypic variation and disease risk. Hum Mol Genet 18(R2):R202-R210.

Nucera C, Muzzi P, Tiveron C, Farsetti A, La Regina F, Foglio B, Shih S-C, Moretti F, Della Pietra L, Mancini F and others. 2010. Maternal thyroid hormones are transcriptionally active during embryo-foetal development: results from a novel transgenic mouse model. Journal of Cellular and Molecular Medicine 14(10):2417-2435.

Priest NK, Wade MJ. 2010. Maternal-Zygotic Epistasis and the Evolution of Genetic Diseases. Journal of Biomedicine and Biotechnology 2010:478732.

Rapley EA, Nathanson KL. 2010. Predisposition alleles for testicular germ cell tumour. Current Opinion in Genetics & Development 20(3):225-230.

Rapley EA, Turnbull C, Al Olama AA, Dermitzakis ET, Linger R, Huddart RA, Renwick A, Hughes D, Hines S, Seal S and others. 2009. A genome-wide association study of testicular germ cell tumor. Nat Genet 41(7):807-811.

For Peer Review

Rice F, Harold GT, Boivin J, Hay DF, van den Bree M, Thapar A. 2009. Disentangling prenatal and inherited influences in humans with an experimental design. Proc Nat Acad Sci 106(7):2464-2467.

SenthamaraiKannan P, Sartor MA, O'Connor KT, Neumann JC, Klyza JP, Jr., Succop PA, Wagner BD, Karyala S, Medvedovic M, Menon AG. 2011. Identification of maternally regulated fetal gene networks in the placenta with a novel embryo transfer system in mice. Physiological Genomics 43(7):317-324.

Sinsheimer JS, Palmer CGS, Woodward JA. 2003. Detecting genotype combinations that increase risk for disease: The maternal-fetal genotype incompatibility test. Genet Epidemiol 24(1):1-13.

Thapar A, Harold G, Rice F, Ge X, Boivin J, Hay D, van den Bree M, Lewis A. 2007. Do intrauterine or genetic influences explain the foetal origins of chronic disease? A novel experimental method for disentangling effects. BMC Med Res Methodol 7:25.

Umbach DM, Weinberg CR. 2000. The use of case-parent triads to study joint effects of genotype and exposure. Am J Hum Genet 66(1):251-261.

Vermeulen SH, Shi M, Weinberg CR, Umbach DM. 2009. A Hybrid Design: Case-Parent Triads Supplemented by Control-Mother Dyads. Genet Epidemiol 33(2):136-144.

Weinberg CR. 1999. Allowing for missing parents in genetic studies of case-parent triads. Am J Hum Genet 64(4):1186-1193.

Weinberg CR, Wilcox AJ, Lie RT. 1998. A log-linear approach to case-parent-triad data: Assessing effects of disease genes that act either directly or through maternal effects and that may be subject to parental imprinting. Am J Hum Genet 62(4):969-978.

Winder NR, Krishnaveni GV, Hill JC, Karat CLS, Fall CHD, Veena SR, Barker DJP. 2011. Placental programming of blood pressure in Indian children. Acta Paediatrica 100:653-660.

For Peer Review

Wolf JB. 2000. Gene interactions from maternal effects. Evolution 54(6):1882-1898.

Zhou JJ, Pelka S, Lange K, Palmer CGS, Sinsheimer JS. 2011. Dissecting Prenatal, Postnatal, and Inherited Effects: ART and Design. Genet Epidemiol 35(6):437-446.

For Peer Review

FIGURE CAPTIONS

Fig. 1. Power (α=0.05) to detect different types of genetic effects with similar strength (relative risk 1.5) using full and restricted models. The restricted model fits the truly simulated effects plus the second locus main effect when there is an interaction involving this locus. Data from simulated model 6 for R and S, model 2 for MxM interaction, model 4 for two-locus MxO interaction and model 9 for single locus MxO interaction. Left panel: 300 full trios; right panel: 1000 full trios. Analyses conducted using Eq.1 unless otherwise indicated. The analysis assumed a dominant genetic model.

Fig. 2. Type-1 error rate for MxM and MxO interactions at nominal level 0.05 with missing parents. Left panel: total sample of 300 families; right panel: total sample of 1000 families. Data from simulated model 1 and analysed using the full model (Eq.1). The analysis assumed a dominant genetic model.

Fig. 3. Power for MxM and MxO interactions at nominal level 0.05 with 10 to 80% missing fathers. Left panel: total sample of 300 families; right panel: total sample of 1000 families. Data from simulated models 7 and 8 and analysed using the full model (Eq.1). The analysis assumed a dominant genetic model.

For Peer Review

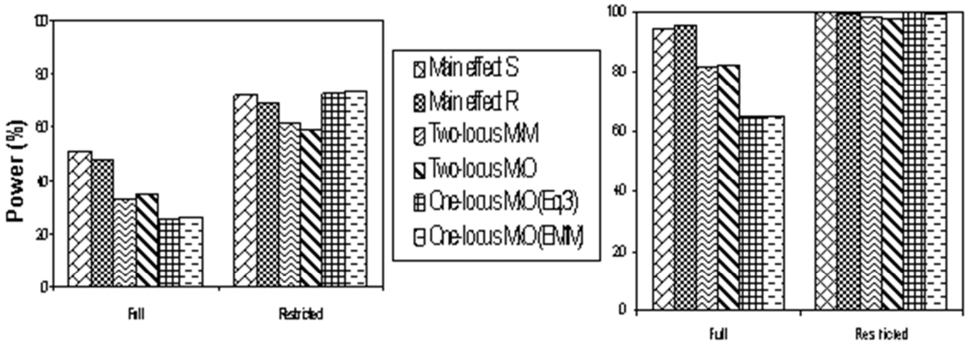

Fig. 1. Power (α=0.05) to detect different types of genetic effects with similar strength (relative risk 1.5) using full and restricted models. The restricted model fits the truly simulated effects plus the second locus main effect when there is an interaction involving this locus. Data from simulated model 6 for R and S, model 2 for MxM interaction, model 4 for two-locus MxO interaction and model 9 for single locus MxO interaction. Left panel: 300 full trios; right panel: 1000 full trios. Analyses conducted using Eq.1 unless otherwise indicated. The analysis assumed a dominant genetic model.
159x59mm (96 x 96 DPI)

eer Review

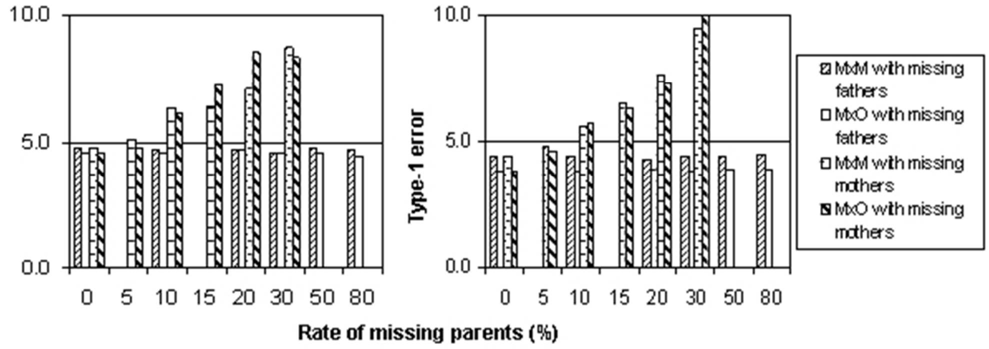

Fig. 2. Type-1 error rate for MxM and MxO interactions at nominal level 0.05 with missing parents. Left panel: total sample of 300 families; right panel: total sample of 1000 families. Data from simulated model 1 and analysed using the full model (Eq.1). The analysis assumed a dominant genetic model.
159x59mm (96 x 96 DPI)

Peer Review

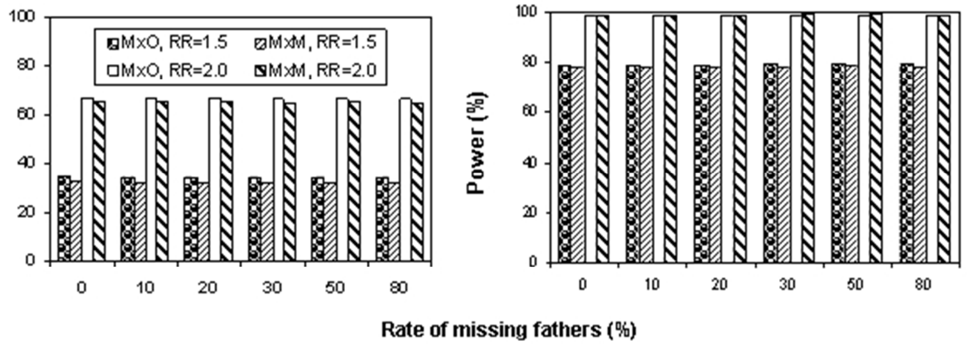

Fig. 3. Power for MxM and MxO interactions at nominal level 0.05 with 10 to 80% missing fathers. Left panel: total sample of 300 families; right panel: total sample of 1000 families. Data from simulated models 7 and 8 and analysed using the full model (Eq.1). The analysis assumed a dominant genetic model.
159x59mm (96 x 96 DPI)

Peer Review

1

TABLE I. Theoretical proportions of family trio types in a two-locus model

Configuration	Mating type	M [a]	F [a]	C [a]	Expected cell proportions		
					M_2=0 [b]	M_2=1 [b]	M_2=2 [b]
1	1	2	2	2	$p_0S_2R_2\mu_1$	$p_1\vartheta_{21}\Phi_{21}S_2R_2\mu_1\tau_1$	$p_2\vartheta_{22}\Phi_{22}S_2R_2\mu_1\tau_2$
2	2	2	1	2	$p_0S_2R_2\mu_2$	$p_1\vartheta_{21}\Phi_{21}S_2R_2\mu_2\tau_1$	$p_2\vartheta_{22}\Phi_{22}\ S_2R_2\mu_2\tau_2$
3	2	2	1	1	$p_0S_2R_1\mu_2$	$p_1\vartheta_{21}\Phi_{11}S_2R_1\mu_2\tau_1$	$p_2\vartheta_{22}\Phi_{12}S_2R_1\mu_2\tau_2$
4	2	1	2	2	$p_0S_1R_2\mu_2$	$p_1\vartheta_{11}\Phi_{21}S_1R_2\mu_2\tau_1$	$p_2\vartheta_{12}\Phi_{22}S_1R_2\mu_2\tau_2$
5	2	1	2	1	$p_0S_1R_1\mu_2$	$p_1\vartheta_{11}\Phi_{11}S_1R_1\mu_2\tau_1$	$p_2\vartheta_{12}\Phi_{12}S_1R_1\mu_2\tau_2$
6	3	2	0	1	$p_0S_2R_1\mu_3$	$p_1\vartheta_{21}\Phi_{11}S_2R_1\mu_3\tau_1$	$p_2\vartheta_{22}\Phi_{12}S_2R_1\mu_3\tau_2$
7	3	0	2	1	$p_0R_1\mu_3$	$p_1\Phi_{11}R_1\mu_3\tau_1$	$p_2\ \Phi_{12}R_1\mu_3\tau_2$
8	4	1	1	2	$p_0S_1R_2\mu_4$	$p_1\vartheta_{11}\Phi_{21}S_1R_2\mu_4\tau_1$	$p_2\vartheta_{12}\ \Phi_{22}S_1R_2\mu_4\tau_2$
9	4	1	1	1	$2p_0S_1R_1\mu_4$	$2p_1\vartheta_{11}\Phi_{11}S_1R_1\mu_4\tau_1$	$2p_2\vartheta_{12}\Phi_{12}S_1R_1\mu_4\tau_2$
10	4	1	1	0	$p_0S_1\mu_4$	$p_1\vartheta_{11}S_1\mu_4\tau_1$	$p_2\vartheta_{12}S_1\mu_4\tau_2$
11	5	1	0	1	$p_0S_1R_1\mu_5$	$p_1\vartheta_{11}\Phi_{11}S_1R_1\mu_5\tau_1$	$p_2\vartheta_{12}\Phi_{12}S_1R_1\mu_5\tau_2$
12	5	1	0	0	$p_0S_1\mu_5$	$p_1\vartheta_{11}S_1\mu_5\tau_1$	$p_2\vartheta_{12}S_1\mu_5\tau_2$
13	5	0	1	1	$p_0R_1\mu_5$	$p_1\Phi_{11}R_1\mu_5\tau_1$	$p_2\Phi_{12}R_1\mu_5\tau_2$
14	5	0	1	0	$p_0\mu_5$	$p_1\mu_5\tau_1$	$p_2\mu_5\tau_2$
15	6	0	0	0	$p_0\mu_6$	$p_1\mu_6\tau_1$	$p_2\mu_6\tau_2$

[a] M, F, C: maternal, paternal and child genotypes at the first locus.

[b] M_2= maternal genotypes at the second locus with frequency p_k, $k=0,1,2$. Genotypes are coded as the number of minor alleles (assumed to increase the risk).

For Peer Review

TABLE II. Simulated dominant models to evaluate the interaction tests

**Model**	R	S	ρ	exp(δ)	ϑ	Φ	**Purpose** [a]
1	1	1	1	1	1	1	Type-1 error of Eq.1 and Eq.2 assuming mating symmetry, Eq.1 assuming HWE, full data and missing parents, robustness to frequency misspecification.
2	1	1	1	1	1.5	1	Power + type-1 error.
3	1	1	1	1	2	1	Power + type-1 error.
4	1	1	1	1	1	1.5	Power + type-1 error.
5	1	1	1	1	1	2	Power + type-1 error.
6	1.5	1.5	1	1	1	1	Type-1 error + power for main effects of child and maternal genotypes, estimation of genotype frequencies with Eq.2
7	1	1	1	1	1.5	1.5	Power comparison eq.1 *vs.* eq.2, full data and missing parents, estimation of genotype frequencies with Eq.2
8	1	1	1	1	2	2	Power comparison eq.1 *vs.* eq.2, full data and missing parents, estimation of genotype frequencies with Eq.2
9	1	1	1.5	1	1	1	Power comparison eq.3 *vs.* EMIM.
10	1	1	2	1	1	1	Power comparison eq.3 *vs.* EMIM.

[a] *R, S, Φ,* ϑ and δ represent respectively the main effect of child locus 1, main effect of maternal locus 1, two-locus MxO interactions, two-locus MxM interactions and maternal second locus main effect. The purpose of simulations was to evaluate the power and/or type-1 error to detect MxM and

For Peer Review

MxO interactions in 300 and 1000 full trios using Eq.1, assuming mating symmetry and correctly specifying locus 2 genotype frequencies, unless otherwise indicated. Dominant models are simulated for all relevant effects, i.e. $R=R_1=R_2$, $S=S_1=S_2$, $\rho=\rho_{11}=\rho_{12}=\rho_{21}=\rho_{22}$, $\vartheta=\vartheta_{11}=\vartheta_{12}=\vartheta_{21}=\vartheta_{22}$ and $\Phi=\Phi_{11}=\Phi_{12}=\Phi_{21}=\Phi_{22}$ and locus 2 has no main maternal effect ($\delta_1=\delta_2=0$). The data simulated in these models were analysed assuming dominant inheritance of all the effects tested and two nuisance parameters δ_1 and δ_2 were fitted for the main maternal effect of the second locus.

For Peer Review

1

TABLE III. Simulated codominant models [a]

Model	R_1	R_2	S_1	S_2	Φ_{11}	Φ_{12}	Φ_{21}	Φ_{22}	ϑ_{11}	ϑ_{12}	ϑ_{21}	ϑ_{22}	δ_1	δ_2
**11+11a**	1	1	1	1	1.3	1.69	1.69	2.197	1	1	1	1	1	1
**12+12a**	1	1	1	1	1	1	1	1	1.4	1.96	1.96	2.744	1	1
**13+13a**	1	1	1	1	1.3	1.69	1.69	2.197	1.4	1.96	1.96	2.744	1	1
**14+14a**	1.3	1.69	1.4	1.96	1	1	1	1	1	1	1	1	1	1

[a] R_j, S_i, Φ_{jk} and ϑ_{ik} and δ_k represent respectively the main effect of child locus 1, main effect of maternal locus 1, two-locus MxO interactions, two-locus MxM interactions and maternal second locus main effect. Models 11-14 were simulated under parental mating symmetry while 11a-14a were simulated under parental mating asymmetry with similar relative risks. Data simulated under these models were analysed using Eq.1 assuming an unconstrained genetic model for all parameters of interest (S_i, R_j, Φ_{jk} and ϑ_{ik}) along with nuisance parameters δ_1 and δ_2. Robustness to genetic model misspecification was assessed by repeating the analysis assuming dominant effects in the analysis.

For Peer Review

1

TABLE IV. Power and type-1 error (%) of the interactions using full trios (α=0.05)

Simulated Model [(a)]	# families	Method [(b)]	Assumed p_k if Eq. 1 [(c)]	ϑ [(d)]	Φ [(d)]
1	300	Eq.1	True	4.8	4.6
1	300	Eq.1	False 1	4.8	4.6
1	300	Eq.1	False 2	4.8	4.6
1	300	Eq.1_hwe	True	4.5	4.7
1	300	Eq.2	-	4.8	4.6
1	1000	Eq.1	True	4.4	3.8
1	1000	Eq.1	False 1	4.4	3.8
1	1000	Eq.1	False 2	4.4	3.8
1	1000	Eq.1_hwe	True	4.2	3.8
1	1000	Eq.2	-	4.4	3.8
6	300	Eq.1	True	5.2	5.7
6	300	Eq.2	-	5.2	5.7
6	1000	Eq.1	True	5.8	5.4
6	1000	Eq.2	-	5.8	5.4
2	300	Eq.1	True	32.7	4.3
3	300	Eq.1	True	73.6	4.5
4	300	Eq.1	True	5.1	34.6
5	300	Eq.1	True	5.2	73.9
2	1000	Eq.1	True	81.9	5.5
3	1000	Eq.1	True	99.5	5.3
4	1000	Eq.1	True	5.7	82.2
5	1000	Eq.1	True	5.4	99.6
7	300	Eq.1	True	31.9	34.4
7	300	Eq.1_hwe	True	32.1	34.4
7	300	Eq.2	-	31.9	34.4
8	300	Eq.1	True	65.0	66.6
8	300	Eq.1_hwe	True	65.9	67.5

For Peer Review

TABLE IV (Continued)

8	300	Eq.2	-	65.0	66.6
7	1000	Eq.1	True	78.2	79.2
7	1000	Eq.1_hwe	True	78.7	79.3
7	1000	Eq.2	-	78.2	79.2
8	1000	Eq.1	True	99.0	99.0
8	1000	Eq.1_hwe	True	99.3	99.0
8	1000	Eq.2	-	99.0	99.0
11	1000	Eq.1	True	4.8	60.6
12	1000	Eq.1	True	84.9	5.0
13	1000	Eq.1	True	85.5	61.0
14	1000	Eq.1	True	4.9	5.1
11a	1000	Eq.1	True	4.5	63.5
12a	1000	Eq.1	True	84.5	4.3
13a	1000	Eq.1	True	85.4	62.3
14a	1000	Eq.1	True	4.0	4.6

(a) Each model was simulated in one run with 300 trios and in a separate run with 1000 trios, each repeated 1000 times, and the data were analysed using the approaches in column 3 in a full model assuming dominant (model 1-8) or codominant inheritance (model 11-14 and 11a-14a).

(b) Eq.1 and Eq.2 assume mating symmetry, Eq.1_hwe assumes HWE at locus 1. Genotype frequencies estimated with equation 2 are supplied in supplementary Table S1.

(c) The true genotype frequency distribution simulated was 0.09/0.42/0.49 for the minor homozygous/heterozygous/major homozygous. False 1 and False 2 distributions assumed in some analyses were respectively 0.01/0.18/0.81 and 0.25/0.50/0.25.

(d) Power or type-1error in same column; see simulated models in Tables II and III. Φ and ϑ are MxO and MxM interactions respectively.

For Peer Review

TABLE V. TGCT family data analysis with Eq.1

Analysis model	Locus 1 [a]	Locus 2 [a]	R (pvalue) [b]	S (pvalue) [b]	Φ (pvalue) [b]	ϑ (pvalue) [b]
Full	*KITLG* / rs995030	*SHBG* / rs6259	**0.41 ($3x10^{-4}$)**	1.05 (0.84)	1.34 (0.56)	1.63 (0.26)
	KITLG / rs1508595		**0.37 ($4x10^{-4}$)**	0.85 (0.55)	1.32 (0.59)	2.19 (0.07)
Restricted to two effects	*KITLG* / rs995030	*SHBG* / rs6259	**0.43 ($2x10^{-4}$)**	-	-	1.84 (0.12)
	KITLG / rs1508595		**0.40 ($2x10^{-4}$)**	-	-	**2.19 (0.03)**

[a] Minor allele frequencies are 0.14 for rs995030, 0.17 for rs1508595 and 0.20 for rs6259.

[b] Estimated relative risks under a dominant model for locus 1 inherited genotype (R), locus 1 maternal genotype (S), two-locus MxO (Φ) and MxM (ϑ) interactions.

For Peer Review

SUPPLEMENTARY DATA

TABLE S1. Second locus genotype frequency estimates from 1000 simulations using Eq.2 in models with mating type symmetry or asymmetry (median and inter-quartile range)

Model	N	p_0 (IQR)	p_1 (IQR)	p_2 (IQR)
6	300	0.489 (0.452, 0.524)	0.422 (0.391, 0.451)	0.090 (0.078, 0.103)
6	1000	0.490 (0.471, 0.510)	0.420 (0.401, 0.438)	0.090 (0.083, 0.097)
7	300	0.490 (0.453, 0.525)	0.421 (0.390, 0.450)	0.090 (0.079, 0.102)
7	1000	0.491 (0.471, 0.508)	0.419 (0.403, 0.436)	0.090 (0.084, 0.096)
8	300	0.488 (0.450, 0.525)	0.423 (0.392, 0.453)	0.090 (0.078, 0.101)
8	1000	0.489 (0.469, 0.511)	0.420 (0.401, 0.438)	0.090 (0.084, 0.096)
13a	1000	0.489 (0.466, 0.513)	0.420 (0.399, 0.445)	0.089 (0.077, 0.101)
14a	1000	0.492 (0.469, 0.516)	0.418 (0.396, 0.441)	0.089 (0.076, 0.103)

The true values are 0.49 for p_0, 0.42 for p_1 and 0.09 for p_2 (MAF 0.3 and Hard-Weinberg equilibrium). This table shows that genotype frequencies estimates are accurate whether there is mating symmetry (models 6-8) or asymmetry (models 13a and 14a) and whether locus 2 has interaction effects (models 7, 8 and 13a) or has no effect at all (models 6 and 14a). The full model was used assuming dominant (models 6-8) or codominant (models 13a and 14a) effects.

For Peer Review

TABLE S2. Risk parameter estimates with full and incomplete trios in dominant models (Eq.1, full model) (median and inter-quartile range)

Simulated model	# families	Missing parent	% missing	Φ [a]	ϑ [a]
2	300	None	0	1.006 (0.855,1.182)	1.485 (1.241, 1.752)
2	1000	None	0	1.005 (0.908,1.103)	1.498 (1.365, 1.651)
4	300	None	0	1.502 (1.263, 1.790)	1.005 (0.841, 1.189)
4	1000	None	0	1.509 (1.371, 1.660)	0.998 (0.910, 1.103)
6	300	None	0	1.004 (0.837, 1.187)	0.993 (0.821, 1.197)
6	1000	None	0	0.995 (0.908, 1.104)	0.995 (0.905, 1.099)
1	300	None	0	1.003 (0.837, 1.177)	0.993 (0.837, 1.186)
1	300	Father	20	1.003 (0.836, 1.175)	0.992 (0.836, 1.187)
1	300	Father	50	1.004 (0.833, 1.178)	0.995 (0.836, 1.188)
1	300	Father	80	1.002 (0.834, 1.177)	0.992 (0.832, 1.186)
7	300	None	0	1.502 (1.254, 1.812)	1.503 (1.246, 1.789)
7	300	Father	20	1.500 (1.255, 1.810)	1.500 (1.245, 1.786)
7	300	Father	50	1.497 (1.253, 1.812)	1.499 (1.242, 1.790)
7	300	Father	80	1.499 (1.256, 1.800	1.496 (1.238, 1.789)
1	1000	None	0	0.994 (0.904, 1.088)	1.003 (0.907, 1.100)
1	1000	Mother	10	0.989 (0.902, 1.090)	1.005 (0.906, 1.106)
1	1000	Mother	20 [b]	0.988 (0.887, 1.097)	1.008 (0.900, 1.125)
7	1000	None	0	1.502 (1.361, 1.662)	1.501 (1.350, 1.649)
7	1000	Mother	10	1.504 (1.347, 1.671)	1.500 (1.355, 1.665)
7	1000	Mother	20 [b]	1.502 (1.339, 1.679)	1.499 (1.335, 1.689)

[a] Φ and ϑ are MxO and MxM interactions respectively. All true values simulated are either 1.0 or 1.5 (see simulated models description in Table II).

[b] Despite correctly estimating these parameters, the test is not valid when 20% mothers are missing. The false positive rates are 0.073 and 0.076 for MxO and MxM interactions respectively, significantly higher than the expected 0.05 (one-sided binomial distribution p-values of 0.001 and 0.0003).

For Peer Review

TABLE S3. Risk parameter estimates with 1000 full trios in unconstrained analysis of datasets simulated with symmetrical parental mating (Eq.1, full model) (median and inter-quartile range)

Parameter [a]	Model 11	Model 12	Model 13	Model 14
Φ_{11}	1.294 (1.171, 1.407)	1.001 (0.906, 1.122)	1.295 (1.167, 1.437)	1.000 (0.896, 1.116)
Φ_{12}	1.682 (1.426, 1.990)	1.007 (0.858, 1.173)	1.671(1.427, 2.004)	1.000 (0.822, 1.217)
Φ_{21}	1.696 (1.417, 2.025)	0.999 (0.831, 1.184)	1.712 (1.430, 2.024)	1.000 (0.859, 1.158)
Φ_{22}	2.126 (1.625, 2.874)	1.000 (0.767, 1.311)	2.195 (1.727, 2.768)	1.001 (0.741, 1.328)
ϑ_{11}	0.996 (0.902, 1.120)	1.404 (1.248, 1.539)	1.392 (1.258, 1.563)	0.997 (0.896, 1.109)
ϑ_{12}	1.004 (0.851, 1.185)	1.945 (1.654, 2.313)	1.981 (1.695, 2.327)	0.995 (0.829, 1.212)
ϑ_{21}	1.000 (0.842, 1.208)	1.991 (1.658, 2.368)	1.957 (1.629, 2.357)	1.003 (0.858, 1.167)
ϑ_{22}	1.004 (0.766, 1.316)	2.719 (2.075, 3.551)	2.750 (2.174, 3.493)	0.992 (0.745, 1.306)

[a] Φ_{ij} and ϑ_{ij} are MxO and MxM interactions respectively.

TABLE S4. Parameter estimates with 1000 full trios in unconstrained analysis of datasets simulated under parental mating asymmetry (Eq.1, full model) (median and inter-quartile range)

Parameter [a]	Model 11a	Model 12a
Φ_{11}	1.306 (1.168, 1.445)	1.001 (0.896, 1.116)
Φ_{12}	1.704 (1.423, 2.026)	0.986 (0.844, 1.164)
Φ_{21}	1.698 (1.433, 1.984)	1.002 (0.854, 1.176)
Φ_{22}	2.179 (1.742, 2.784)	0.985 (0.785, 1.251)
ϑ_{11}	0.994 (0.901, 1.104)	1.403 (1.257, 1.559)
ϑ_{12}	1.001 (0.852, 1.168)	1.992 (1.679, 2.315)
ϑ_{21}	0.982 (0.828, 1.194)	1.970 (1.647, 2.348)
ϑ_{22}	1.011 (0.770, 1.301)	2.727 (2.140, 3.542)

[a] Φ_{ij} and ϑ_{ij} are MxO and MxM interactions respectively.

For Peer Review

TABLE S5. Parameter estimates using 1000 families with and without missing fathers in unconstrained analysis of datasets simulated under parental mating asymmetry (Eq.1, full model) (median and inter-quartile range)

Model	Estimated Parameter [a]	Full trios	50% missing	80% missing
13a	Φ_{11}	1.302 (1.152, 1.453)	1.302 (1.151, 1.454)	1.300 (1.148, 1.457)
	Φ_{12}	1.706 (1.451, 2.030)	1.704 (1.449, 2.037)	1.713 (1.452, 2.030)
	Φ_{21}	1.691 (1.426, 1.988)	1.691 (1.423, 1.986)	1.686 (1.423, 1.985)
	Φ_{22}	2.186 (1.770, 2.779)	2.191 (1.763, 2.776)	2.187 (1.764, 2.773)
	ϑ_{11}	1.394 (1.250, 1.560)	1.394 (1.247, 1.558)	1.394 (1.249, 1.561)
	ϑ_{12}	1.957 (1.678, 2.298)	1.954 (1.672, 2.296)	1.955 (1.680, 2.295)
	ϑ_{21}	1.972 (1.643, 2.378)	1.972 (1.647, 2.377)	1.973 (1.644, 2.373)
	ϑ_{22}	2.757 (2.195, 3.524)	2.754 (2.185, 3.518)	2.743 (2.199, 3.520)
14a	Φ_{11}	0.998 (0.887, 1.112)	0.992 (0.884, 1.118)	0.990 (0.884, 1.117)
	Φ_{12}	0.993 (0.815, 1.212)	0.994 (0.827, 1.206)	0.997 (0.824, 1.207)
	Φ_{21}	0.997 (0.863, 1.159)	0.995 (0.858, 1.155)	0.994 (0.855, 1.155)
	Φ_{22}	0.990 (0.760, 1.308)	0.983 (0.761, 1.265)	0.981 (0.758, 1.273)
	ϑ_{11}	1.008 (0.902, 1.116)	1.008 (0.901, 1.111)	1.009 (0.902, 1.111)
	ϑ_{12}	0.999 (0.837, 1.207)	1.003 (0.840, 1.219)	1.003 (0.839, 1.219)
	ϑ_{21}	0.996 (0.861, 1.174)	0.989 (0.855, 1.157)	0.989 (0.856, 1.154)
	ϑ_{22}	1.003 (0.771, 1.300)	1.006 (0.769, 1.308)	1.007 (0.768, 1.306)

[a] Φ_{ij} and ϑ_{ij} are MxO and MxM interactions respectively.

For Peer Review

All risk parameters estimates (Tables S2-S5) are close to their true values (see Tables II and III for more details on the simulated models). As expected, the inter-quartile range (IQR) is wider in smaller datasets (Table S2) and for any sample size, the IQR widens as the number of minor alleles increases, as there are fewer observations in those categories (Table S3-S5). Table S5 further shows that missing fathers up to 80% do not affect the estimation accuracy or precision.

TABLE S6. Estimated power and type 1 error (%) for dominant or codominant main effects of one locus in full models assuming parental mating symmetry in the analysis when symmetry or asymmetry was simulated (1000 simulations, α=0.05)

Simulated model	# Families	Method	Maternal effect (S)	Offspring effect (R)
6	300	Eq.2	51.0	47.8
6	300	Eq.1	51.0	47.8
6	300	Eq.1_hwe	55.6	51.9
6	1000	Eq.2	94.5	95.3
6	1000	Eq.1	94.5	95.3
6	1000	Eq.1_hwe	96.7	95.9
13	1000	Eq.1	4.6	4.7
13a	1000	Eq.1	100	4.9

Similarly to the interactions, Eq.1 and Eq.2 are equivalent for the detection of the main effects of locus 1. As has been reported previously (Sinsheimer et al., 2003), when parental mating is asymmetrical, assuming symmetry in the analysis inflates type-1 error rate of the main maternal effect (type 1 error of 100% in model 13a), but the test remains valid for the main child effect and two-locus interactions (see also table IV in the main text, model 11a, 12a and 14a).

For Peer Review

TABLE S7. Impact of misspecifying the second locus genotype frequencies on the validity to test its main effect in a full model (1000 simulations, α=0.05)

# Families	Assumed p_k [a]	Type-1 error (%)
300	False 1	40.4
300	True	5.0
300	False 2	36.5
1000	False 1	90.4
1000	True	5.1
1000	False 2	88.9

[a] The true simulated genotype distribution was 0.09/0.42/0.49 for minor homozygous/ heterozygous/major homozygous and analyses assumed either this true distribution or 0.0625/0.5625/0.375 (False 1) or 0.1225/0.4225/0.455 (False 2). The main effect of the second locus has a correct test size when the correct genotype is specified. However, small deviations from true values invalidate the test. Data from simulated model 1.

TABLE S8. Power (%) to detect the interactions at nominal 5% level using 1000 families of which some mothers are ungenotyped at both loci (1000 simulations, full model)

Simulated	Missing mothers (%)	ϑ [a]	Φ [a]
7	0	78.2	79.2
7	5	78.2	78.9
7	10	78.7	78.1
8	0	99.0	99.0
8	5	99.2	99.0
8	10	98.6	99.1

[a] Φ and ϑ are MxO and MxM interactions respectively. This table shows that power is unaffected by up to 10% missing mothers.

For Peer Review

TABLE S9. Power and type 1 error for two-locus interactions at nominal 5% level with 1000 families including missing fathers when mating asymmetry is simulated but the symmetry is assumed in the analysis (Full model)

Missing fathers (%)	Simulated model	ϑ [a]	Φ [a]
0	13a	85.6	62.4
20	13a	85.6	62.6
50	13a	86.1	62.6
80	13a	85.4	62.3
0	14a	4.0	4.6
20	14a	3.9	4.7
50	14a	4.1	4.7
80	14a	4.1	4.6

[a] Φ and ϑ are MxO and MxM interactions respectively. This table illustrates that when parental mating types are asymmetrical and up to 80% fathers are not genotyped, the MxM and MxO interactions tests have non-inflated type 1 error and similar power as in full trios in symmetrical or asymmetrical mating designs (see also main text Table IV, model 13).

For Peer Review

TABLE S10. Robustness to model misspecification: power and type I error of Eq.1 assuming a dominant mode of action when codominance was simulated (full trios and some missing fathers with a total of 1000 families in 1000 simulations)

Simulated Model	% missing fathers	ϑ [a]	Φ [a]
11	0	4.7	81.1
12	0	89.2	5.2
13	0	93.7	81.3
14	0	5.4	5.0
13	50	93.3	80.1
13	80	92.8	78.9
14	50	4.9	3.7
14	80	4.9	3.8

[a] ϑ and Φ are respectively MxM and MxO interactions. When a dominant model is wrongly assumed, type I error remains close to its expected value of 5% for all simulated null effects (see Table III for simulated models), while the power is higher than in previous analyses (Table IV, models 11-14) where each interaction was a composite effect of 4 parameters. Full model used.

For Peer Review

TABLE S11. Risk parameter estimates under a misspecified genetic model: Analysis assuming dominant interactions when codominance was simulated (Full trios and 80% missing fathers, 1000 simulations) (median and inter-quartile range)

Simulated model	% missing father	ϑ [a]	Φ [a]
13	0	1.660 (1.509, 1.831)	1.501 (1.371, 1.654)
14	0	0.994 (0.898, 1.094)	1.005 (0.910, 1.105)
13	80	1.645 (1.502, 1.830)	1.502 (1.366, 1.650)
14	80	1.009 (0.912, 1.109)	1.000 (0.907, 1.100)

[a] ϑ and Φ are respectively MxM and MxO interactions. This table illustrates that, as expected, when a dominant model is wrongly assumed, the accuracy of parameters estimation is limited if the effect exists. True parameter values are given in Table III. Full model used.

REFERENCES

Sinsheimer JS, Palmer CGS, Woodward JA. 2003. Detecting genotype combinations that increase risk for disease: The maternal-fetal genotype incompatibility test. Genetic Epidemiology 24(1):1-13.

For Peer Review
